# The divergent ER-mitochondria encounter structures (ERMES) are conserved in parabasalids but lost in several anaerobic lineages with hydrogenosomes

**DOI:** 10.1186/s12915-023-01765-1

**Published:** 2023-11-15

**Authors:** Jitka Kučerová, Alois Zdrha, Abhishek Shinde, Karel Harant, Ivan Hrdý, Jan Tachezy

**Affiliations:** 1 Department of Parasitology, Faculty of Science, Charles University, BIOCEV, Průmyslová 595, 25242 Vestec, Czech Republic; 2 OMICS Proteomics Laboratory, Faculty of Science, Charles University, BIOCEV, Průmyslová 595, 25242 Vestec, Czech Republic

**Keywords:** ERMES, *Trichomonas vaginalis*, Hydrogenosomes, Endoplasmic reticulum, Anaerobiosis, Structure, Cardiolipin

## Abstract

**Background:**

The endoplasmic reticulum (ER)-mitochondria membrane contact sites (MCS) are extensively studied in aerobic eukaryotes; however, little is known about MCS in anaerobes with reduced forms of mitochondria named hydrogenosomes. In several eukaryotic lineages, the direct physical tether between ER and the outer mitochondrial membrane is formed by ER-mitochondria encounter structure (ERMES). The complex consists of four core proteins (Mmm1, Mmm2, Mdm12, and Mdm10) which are involved in phospholipid trafficking. Here we investigated ERMES distribution in organisms bearing hydrogenosomes and employed *Trichomonas vaginalis* as a model to estimate ERMES cellular localization, structure, and function.

**Results:**

Homology searches revealed that Parabasalia-Anaeramoebae, anaerobic jakobids, and anaerobic fungi are lineages with hydrogenosomes that retain ERMES, while ERMES components were gradually lost in Fornicata, and are absent in Preaxostyla and Archamoebae. In *T. vaginalis* and other parabasalids, three ERMES components were found with the expansion of Mmm1. Immunofluorescence microscopy confirmed that Mmm1 localized in ER, while Mdm12 and Mmm2 were partially localized in hydrogenosomes. Pull-down assays and mass spectrometry of the ERMES components identified a parabasalid-specific Porin2 as a substitute for the Mdm10. ERMES modeling predicted a formation of a continuous hydrophobic tunnel of TvMmm1-TvMdm12-TvMmm2 that is anchored via Porin2 to the hydrogenosomal outer membrane. Phospholipid-ERMES docking and Mdm12-phospholipid dot-blot indicated that ERMES is involved in the transport of phosphatidylinositol phosphates. The absence of enzymes involved in hydrogenosomal phospholipid metabolism implies that ERMES is not involved in the exchange of substrates between ER and hydrogenosomes but in the unidirectional import of phospholipids into hydrogenosomal membranes.

**Conclusions:**

Our investigation demonstrated that ERMES mediates ER-hydrogenosome interactions in parabasalid *T. vaginalis*, while the complex was lost in several other lineages with hydrogenosomes.

**Supplementary Information:**

The online version contains supplementary material available at 10.1186/s12915-023-01765-1.

## Background

Interorganellar membrane contact sites (MCS) play a fundamental role in organellar biogenesis and homeostasis. In MCS, the membranes interact via a protein-lipid or protein–protein tether that keeps the membranes in close proximity. The most studied interactions involve the endoplasmic reticulum (ER) and mitochondria. In yeast such as *Saccharomyces cerevisiae* and *Zygosaccharoyces rouxii*, ER is tethered to the mitochondrial outer membrane (MOM) by a protein complex named ER-mitochondria encounter structure (ERMES), which facilitates a non-vesicular exchange of phospholipids [1–3]. In addition, multiple other functions have been attributed to ERMES including regulation of mitochondrial morphology [4], mitochondrial DNA inheritance [5], mitophagy [6], mitochondrial protein import [7, 8], and Ca^2+^ homeostasis [9]*.* ERMES consists of four core proteins, maintenance of mitochondrial morphology 1 (Mmm1), Mmm2 (syn. mitochondrial distribution and morphology 34, Mdm34), Mdm10, and Mdm12. Mmm1 possesses an N-terminal transmembrane domain (TMD) anchoring the protein to the membrane of ER [8]. Mmm2 is associated with the mitochondrial outer membrane (MOM), and both proteins bind to cytosolic Mdm12 that bridges ER and MOM subunits. All three proteins contain synaptotagmin-like mitochondrial lipid-binding protein (SMP) domains that form a hydrophobic moiety for phospholipid transfer [10]. Mmm1 and Mmm2 possess long flexible linkers that were proposed to tether the complex to ER and MOM membranes and enable its movement for lipid transfer [2]. Mmm1 forms a homodimer [3, 8], which is flanked by two Mdm12 subunits [3]; however, little is known about Mmm2 interactions within the complex. Mdm10 is a β-barrel protein located in MOM [11] that binds Mmm2 to anchor the ERMES complex in MOM [1]. In addition to core ERMES subunits, several proteins such as Gem1, Tom7, Arf1, and Erm1 were implicated in the regulation of ERMES function [12–14].

ERMES was initially proposed to be a strictly fungal complex that arose as an evolutionary innovation in this lineage. However, later searches across eukaryotic phyla identified ERMES orthologs in Amoebozoa, Discoba, Glaucophyta, and Metamonada, while metazoans, Stramenopiles-Alveolata-Rhizaria (SAR), Chlorophyta, and Rhodophyta seem to be devoid of ERMES [15]. What is the evolutionary scenario behind the observed patchy distribution of ERMES is not clear; however, the simplest explanation is that ERMES was present in the last eukaryotic common ancestor (LECA) and subsequently was replaced by other tethering factors or lost in multiple eukaryotic lineages [15]. Intuitively, the losses of ERMES could be expected in anaerobic protists with reduced forms of mitochondria such as hydrogenosomes and mitosomes (reviewed in Tachezy 2019) [16, 17]. Both hydrogenosomes and mitosomes lost organellar genomes, and most typical metabolic functions including the tricarboxylic acid cycle, respiratory complexes, and F_O_F_1_-ATP synthase. In the hydrogenosomes, ATP is synthesized by substrate-level phosphorylation while energy metabolism is absent in mitosomes.

Hydrogenosomes and mitosomes are spherical organelles limited with double membranes without cristae [18] that morphologically remind ball-like mitochondria of yeast with deleted genes for ERMES subunits [4, 19–21]. Indeed, ERMES components have not been identified in the genomes of any organisms with mitosomes including *Giardia intestinalis* (Metamonada), *Entamoeba histolytica* (Amoebozoa), and microsporidia (Fungi) [15]. In contrast, genes with homology to ERMES components have been found in hydrogenosome-bearing anaerobic fungus *Piromyces* sp., and three putative ERMES components of unclear subunit classification were predicted also in *Trichomonas vaginalis* (Metamonada) [15]. These predictions raise the question of whether ERMES operates in ER-hydrogenosome interactions and whether the formation of ERMES is a common feature for hydrogenosomes, which discriminate these organelles from mitosomes [22].

Metamonada is a particularly attractive group of unicellular eukaryotes (protists) to study the evolution and function of ERMES. It is represented by protists with exclusively anaerobic metabolism including free-living, symbiotic, and parasitic species with various forms of mitochondria adapted to anaerobiosis. They are sorted into four major lineages: Parabasalia (e.g., *T. vaginalis*) with hydrogenosomes; Fornicata such as *Spironucleus salmonicida* and *G. intestinalis* with hydrogenosomes and mitosomes, respectively [23, 24]; Preaxostyla which includes species that entirely lost mitochondria (*Monocercomonoides exilis*) [25]; and recently characterized Anaeramoebae (e.g., *Anaeramoeba flamelloides*), with hydrogenosomes that retained some more mitochondrial functions in comparison to Parabasalids [26]. To elucidate how ERMES status correlates with the type of anaerobic form of mitochondria, we performed exhaustive searches for ERMES components across eukaryotes with a particular focus on Metamonada. Next, we experimentally investigated ERMES components in *T. vaginalis* as a model metamonad and an important human parasite. We established the cellular localization of ERMES and constructed ERMES interactome. Based on structural modeling, we propose the formation of a tube-like structure with the hydrophobic surface of the channel and unidirectional transport of phospholipids via ERMES from ER to the hydrogenosomal membrane.

## Results

### ERMES in Parabasalia revealed the expansion of Mmm1

Reciprocal reverse homology searches were used for identifications of ERMES components [27]. In *T. vaginalis*, we found five paralogs of Mmm1, three of them possess a typical N-terminal TMD, which were named TvMmm1a (TVAG_214860), TvMmm1b (TVAG_302900), and TvMmm1c (TVAG_171680), while other two paralogs lack TMDs, and were named TvMmm1d (TVAG_194830), and TvMmm1e (TVAG_139550). The other components included two Mmm2 paralogs (TvMmm2a, TVAG_217400; and Mmm2b TVAG_375920), and Mdm12 (TvMdm12, TVAG_063000). All components possess SMP domain with 3–4 α-helixes and 4–6 β-strands, although the amino acid (AA) sequences of all *T. vaginalis* ERMES components were highly divergent with low similarity (from 18.7 to 32.2%) to yeast sequences (Additional file 1: Table S1). The protein sequence alignment to yeast orthologs revealed that TvMmm1 and TvMmm2 proteins are considerably shorter at N-terminal and C-terminal unstructured domains (tethering loops), respectively (Additional file 2: Fig. S1). TvMmm1 proteins contain a short N-terminal sequence of 1–5 residues and a short linker (22–26 residues) between TMD, and the first common α-helix (c-α1, Additional file 2: Fig. S1A). In comparison, *Z. rouxii* Mmm1 contains N-terminal sequence of 110 residues preceding TMD, a longer flexible linker (54 residues), and an extra α-helix (s-α1, Additional file 2: Fig. S1A). The TvMmm1 paralogs also have a considerably shorter loop (5–6 residues) between c-β2 and c-β3-strands in comparison to the yeast Mmm1 (26 residues). The C-terminal unstructured domains of TvMmm2a and TvMmm2b consist of 12 residues, while *Z. rouxii* contains a long linker of 216 residues (Additional file 2: Fig. S1C). The HMMER searches for Mdm10 in *T. vaginalis* genome were negative.

**Fig. 1:**
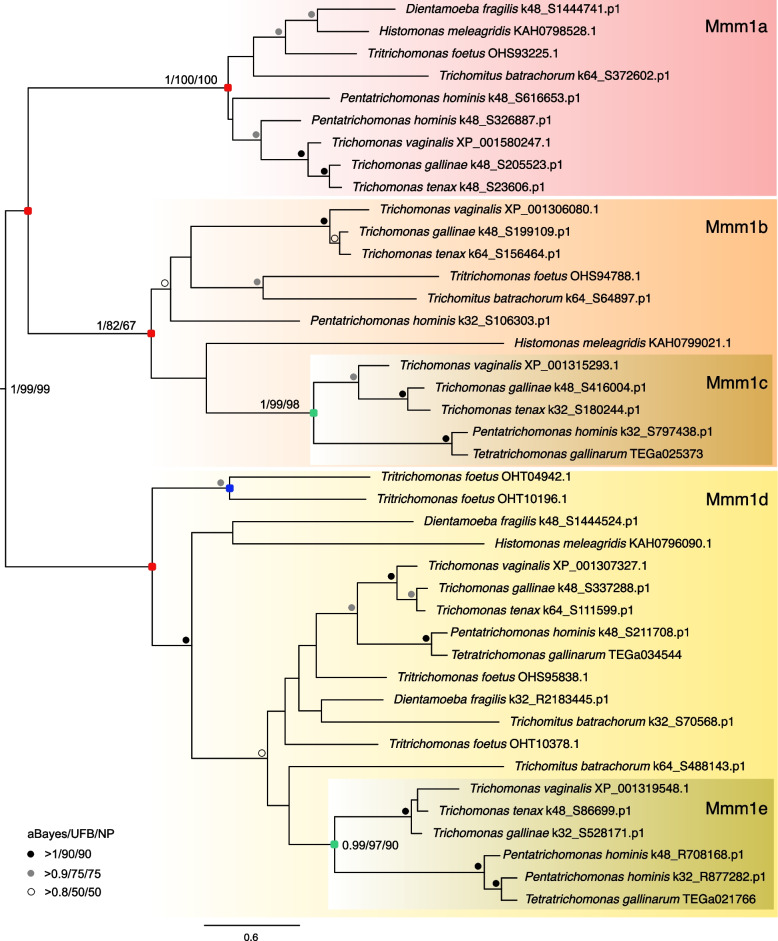
Phylogenetic analysis of Mmm1 expanded paralogs identified in Parabasalia. The maximum likelihood (ML) was constructed using IQ-TREE (Best fit; Q.pfam + F + I + G4 model) with 41 sequences and 218 sites. Red squares represent ancient duplication events; green squares represent Trichomonadea-specific Mmm1 duplicated branches. The support values provide aBayes posterior probability/ML ultra-fast bootstrapping/ML Non-parametric bootstrapping

The number of Mmm1 paralogs in *T. vaginalis* prompted us to extend our searches for ERMES to other parabasalids (Additional file 3: Table S2). Three core ERMES components (Mmm1, Mmm2, and Mdm12) were identified in all members of Trichomonadea (*Trichomonas* species, *Tetratrichomonas gallinarum*, and *Pentatrichomonas hominis*), Tritrichomonadea (*Tritrichomonas foetus*, *Histomonas melleagridis*, and *Dientamoeba fragilis*), and Hypotrichomonadea (*Trichomitus batrachorum*) lineages with Mmm1 being present in multiple copies in all species. The phylogenetic analysis of Mmm1 paralogs revealed that each type of Mmm1 formed a distinct cluster (Fig. 1). Mmm1a, Mmm1b, and Mmm1d were identified in all parabasalid species, which suggests that these three paralogs were present in a common parabasalid ancestor. Mmm1c and Mmm1e seem to derive from Mmm1b and Mmm1d, respectively, only in the Trichomonadea lineage. Of note, the alignment of Mmm1d and Mmm1e proteins revealed that N-terminal TMD is absent only in *T. vaginalis* orthologs (Additional file 2: Fig. S2). The expansion of Mmm1 was not found in *Anaeroamoeba flamelloides*, a free-living relative of Parabasalia. This organism possesses the standard ERMES set including Mdm10 and a single Mmm1.

### ERMES was lost in several lineages with hydrogenosomes

**Fig. 2:**
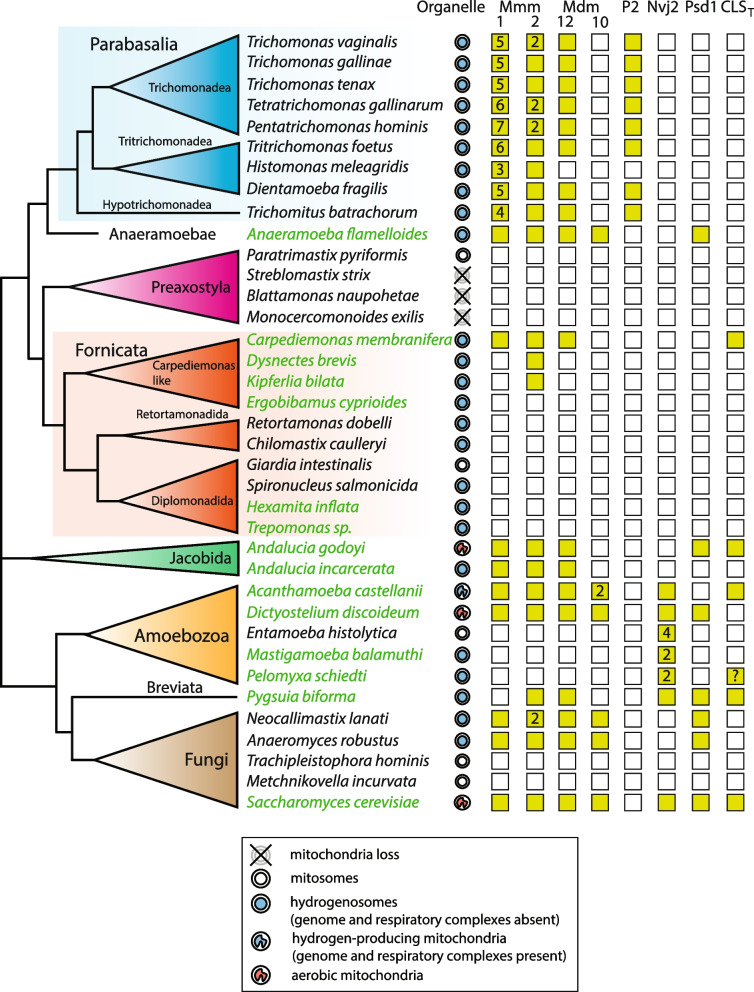
Distribution of ERMES subunits and key enzymes of mitochondrial phospholipid metabolism in anaerobic protists with examples of aerobic relatives. Green and empty squares indicate the presence or absence of the gene. Numbers indicate the number of paralogs. Green names highlight free-living species. Classification is based on homology searches, cell localization predictions, and phylogenetic analysis (Additional file 3: Table S2, Fig. 9, Additional file 2: Fig. S3 and S4). The question mark indicates a protein with inconclusive classification. P2, Porin2; Nvj2, nucleus–vacuole junction 2 protein; Psd1, phosphatidylethanolamine decarboxylase; CLS_T_, cardiolipin synthase with transferase mechanism [29]

Unlike in Parabasalia, searches for ERMES components were negative in parasitic metamonads with hydrogenosomes (*S. salmonicida*, *Chilomastix caulleryi*, *Retortamonas dobelli*) that belong to Fornicata (Fig. 2). However, in free-living fornicates, we found a gradual reduction of ERMES from basal fornicate *Carpediemonas membranifera* that possesses Mmm1 and Mmm2, via *Kipferlia bialata* and *Dysnectes brevis* with only Mmm2 to *Ergobibamus cyprinoides*, in which no ERMES component was identified (Fig. 2). When we extended our searches for protists with hydrogenosomes in other eukaryotic lineages (Fig. 2, Additional file 3: Table S2), no genes coding for ERMES components were found in Archamoebae (*Mastigamoeba balamuthi*, and *Pelomyxa schiedti*), while ERMES was present in their aerobic relatives with mitochondria such as *Dictyostelium discoideum*. Archamoebae possess only a single protein with the SMP domain, Nvj2 (nucleus–vacuole junction 2 protein) [28] that was distinguished from ERMES by phylogenetic analysis (Additional file 2: Fig. S3). In contrast, ERMES-specific components were identified in anaerobic jakobid *Andalucia incarcerata*, anaerobic fungi *Neocallimastix lanati*, and *Anaeromyces robustus*, and a breviate *Pygsuia biforma*. These results indicate that the absence or presence of ERMES is a lineage-specific feature and its losses are not directly linked with the hydrogenosome-to-mitosome transition.

### Cellular localization of ERMES components in *T. vaginalis*

**Fig. 3:**
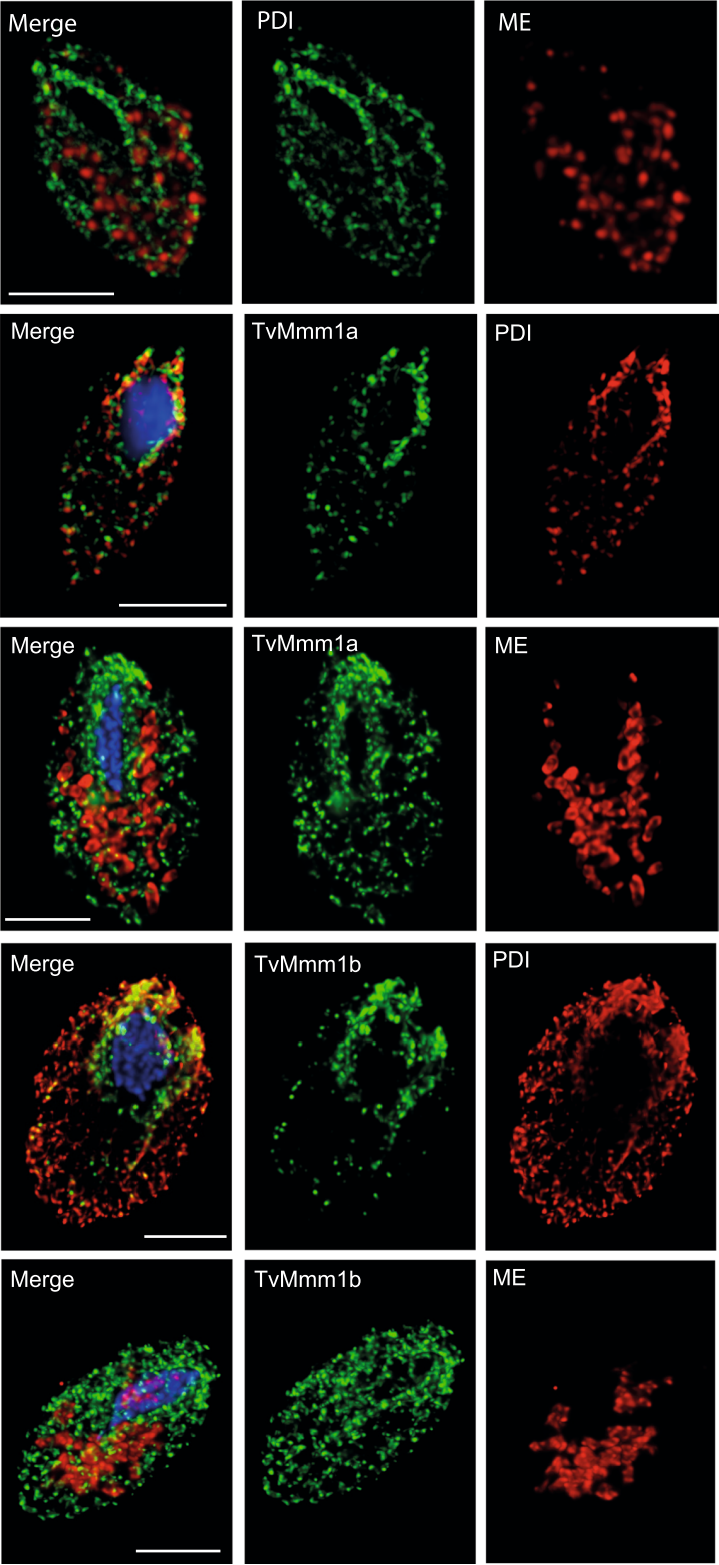
Cellular localization of *T. vaginalis* TvMmm1 observed by confocal microscopy. Hemagglutinin (HA)-tagged recombinant proteins, TvMmm1a, TvMmm1b, and disulfide isomerase (PDI, ER marker) were visualized using mouse α-HA antibody (green). The malic enzyme (ME), a hydrogenosomal marker, was visualized with rabbit α-malic enzyme (red) antibody. Alternatively, HA-tagged recombinant TvMmm1a or TvMmmb were co-expressed with recombinant V5-tagged PDI and visualized using mouse α-HA (green) and rabbit α-V5 antibodies (red). The nucleus was stained with DAPI (blue). Bar = 5 μm

**Fig. 4:**
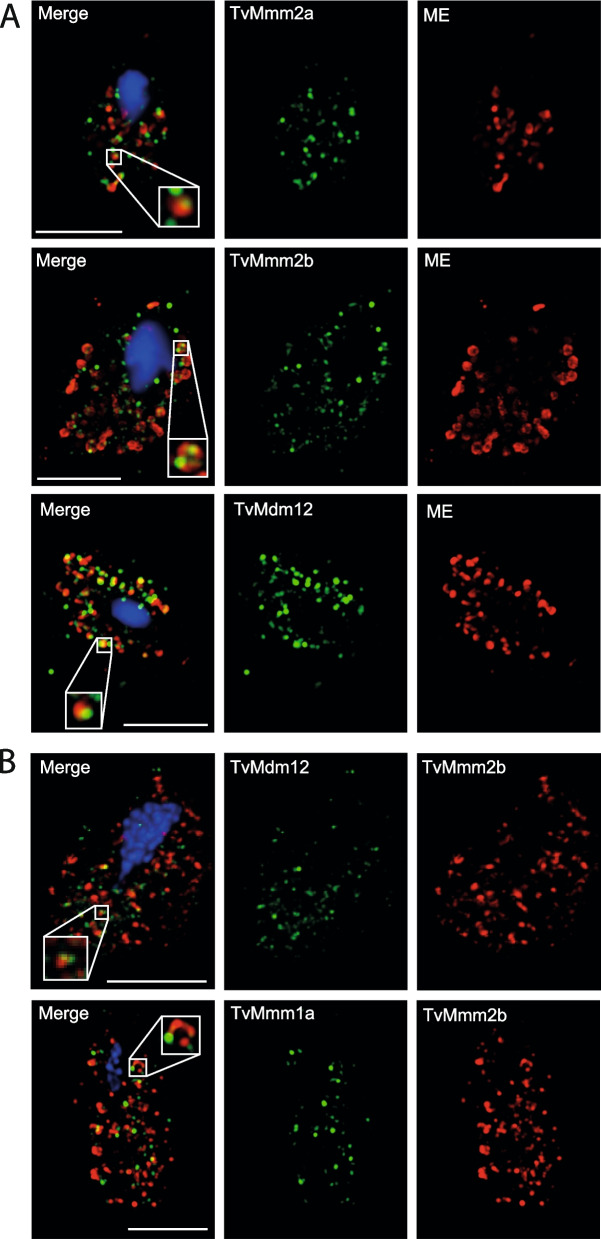
Cellular localization of *T. vaginalis* ERMES components observed by confocal microscopy. **A** Colocalization of TvMmm2a, TvMmm2b, and TvMdm12 with hydrogenosomal malic enzyme (ME). Hemagglutinin (HA)-tagged TvMmm2a, TvMmm2b, and TvMdm12 were visualized using mouse α-HA antibody (green). The malic enzyme (ME) was visualized with rabbit α-malic enzyme (red) antibody. **B** Interactions of TvMmm2b with TvMdm12 and TvMmm1a. HA-tagged TvMdm12 or TvMmm1a were co-expressed with recombinant V5-tagged TvMmm2b and visualized using mouse α-HA antibody (green) and rabbit α-V5 antibody (red). The nucleus was stained with DAPI (blue). Bar = 5 μm

The sequence diversity and absence of tethering loops in TvMmm1 and TvMmm2 proteins prompted us to elucidate the cellular localization of all ERMES components and their possible role in MCS formation. Structured illumination microscopy (SIM) visualization of hydrogenosomes, and ER in *T. vaginalis* using marker proteins malic enzyme and PDI, respectively showed that hydrogenosomes are surrounded by the tubular structures of a rich ER network (Fig. 3). HA-tagged TvMmm1a and TvMmm1b labeled a ring-like structure around the nucleus and numerous small spots scattered within the cytosol (Fig. 3) that co-localized with PDI (PCC *r* = 0.54 and 0.65, respectively). When hydrogenosomes were visualized, a punctate pattern of TvMmm1a and TvMmm1b that rarely co-localized with hydrogenosomal malic enzyme (PCC *r* = 0.017 and 0.006, respectively) was observed (Fig. 3). We cannot exclude the possibility that the strong signal for Mmm1 proteins in ER might be due to their overexpression under the control of a strong promotor, which partially masks their co-localization with hydrogenosomes. In contrast, TvMmm2a and TvMmm2b did localize to hydrogenosomes with a similar, punctate pattern (Fig. 4, PCC *r* = 0.43 and 0.41, respectively). While the malic enzyme was distributed evenly within hydrogenosomes, TvMmm2a and TvMmm2b were labeled as smaller spots at the periphery of the organelles. TvMdm12 colocalized with a malic enzyme in hydrogenosomes with PCC *r* = 0.44. Co-expression of TvMdm12 and TvMmm2b revealed partial co-localization of these proteins or their proximity (PCC *r* = 0.36, Fig. 4). Co-expression of TvMmm1a and TvMmm2b revealed rare but detectable co-localization of these two ERMES components (PCC *r* = 0.15, Fig. 4). Such a limited colocalization supports more kiss-and-run model of interactions between these components than their stable association.

**Fig. 5:**
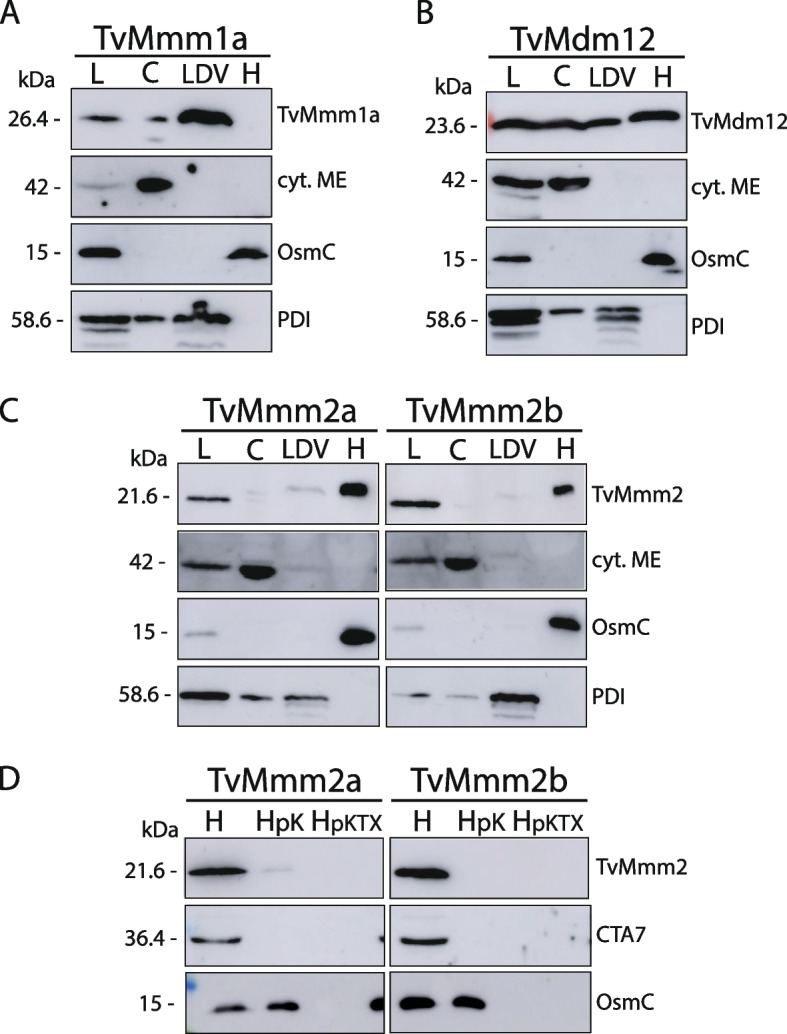
Western blot analysis of *T. vaginalis* ERMES components in subcellular fractions. *T. vaginalis* cells expressing **A** TvMmm1a, **B** TvMdm12, **C** TvMmm2a, or TvMmm2b were sonicated and subcellular fractions were separated using differential and Percoll gradient centrifugation. L, cell lysate; C, cytosolic fraction; LDV, low-density vesicles; H, hydrogenosomal fraction. TvMmm1a, TvMmm2a/b, and TvMdm12 were detected using mouse α-HA antibodies. Cyt. ME, cytosolic malic enzyme (cytosolic marker); OsmC (hydrogenosomal marker); and PDI (ER marker) were visualized by mouse α-cytosolic ME, rat α-OsmC, and rat α-PDI polyclonal antibodies, respectively. **D** TvMmm2 topology test. TvMmm2a and TvMmm2b hydrogenosomal fraction (H) was treated with proteinase K (HpK) and proteinase K with Triton X-100 (HpKTX). TvMmm2a and TvMmm2b were detected by mouse α-HA antibody, OsmC was detected by rat polyclonal α- OsmC antibody, and C-tail anchored protein 7 (CTA7, outer hydrogenosomal membrane marker protein) was detected by rat polyclonal α-CTA7 antibody

Cell localization of ERMES components observed by fluorescence microscopy was corroborated by analysis of subcellular fractions (Fig. 5). Homogenates of cells expressing HA-tagged proteins were used for differential and Percoll gradient centrifugation that resulted in the separation of hydrogenosomes, light density fraction (LDV) that contains ER vesicles [30] and the cytosol (Fig. 5). The strongest signal for TvMmm1a was observed in LDV fraction (Fig. 5A), while TvMmm2a and TvMmm2b were associated with the hydrogenosomes (Fig. 5C). To investigate the topology of TvMmm2a and TvMmm2b in hydrogenosomes, we performed a protease protection assay (Fig. 5D). Hydrogenosomes isolated from *T. vaginalis* cells that expressed TvMmm2a and TvMmm2b were incubated with proteinase K, which resulted in the disappearance of TvMmm2 proteins. The C-tail anchored protein 7 (CTA7) was used as an outer membrane control protein [30]. The protease K did not digest Osmotically inducible protein (OsmC) in the hydrogenosomal matrix, which was membrane-protected and was digested only upon the addition of detergent Triton X-100 (Fig. 5D). The signal for TvMdm12 was similar in all three fractions which is consistent with its function to bridge TvMmm1 and TvMmm2 proteins in MCS (Fig. 5B). Altogether, the cellular localization of all tested proteins is consistent with their predicted function as ERMES components.

### ERMES interactome

To study interacting partners of ERMES components, we co-immunoprecipitated (coIP) protein complexes from *T. vaginalis* lysates using HA-tagged TvMmm1a, TvMmm1b, TvMmm2a, TvMmm2b, and TvMdm12 as baits. Samples were analyzed by label-free quantitative mass spectrometry (LFQ MS) and acquired data were statistically evaluated to identify coIP proteins that were significantly enriched in cells expressing bait in comparison to the control cells (Additional file 4: Table S3, Additional file 2: Fig. S4).

**Fig. 6:**
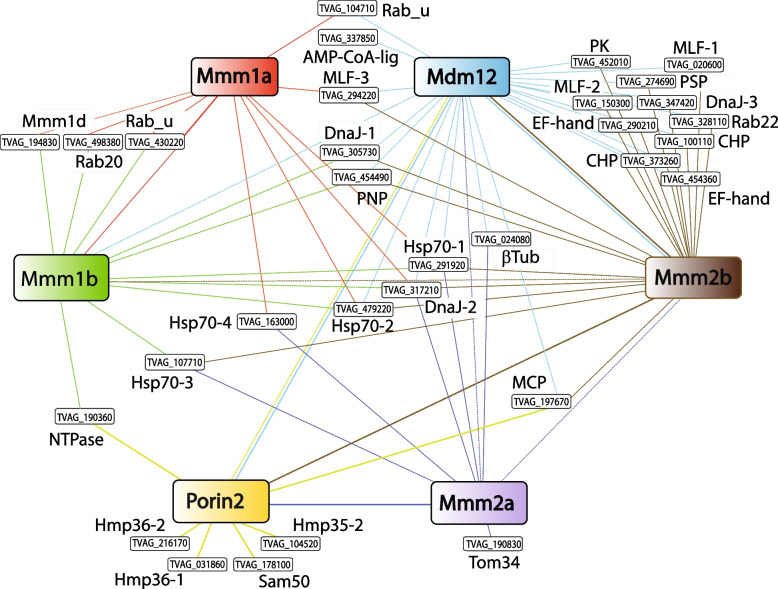
Interactome of *T. vaginalis* ERMES components. The interactome was constructed based on coIP experiments and mass spectrometry (Additional file 4: Table S3). Large colored boxes indicate the baits that were used for the coIP complexes. CoIP proteins are linked with color-coded lines. Solid lines indicate significantly enriched proteins coIP with the bait (FDR 0.05, S = 1), and dotted lines indicate ERMES components that coIP under the threshold of significance. Proteins that coIP with more than a single bait are included, except selected proteins of interest that coIP with Porin2 and Mmm2a baits. AMP-CoA-lig, AMP CoA ligase; CHP, conserved hypothetical protein; EF-hand, EF-hand calcium-binding protein; MCP, mitochondrial carrier protein; MLF, myeloid leukemia factor; NTPase, P-loop containing nucleoside triphosphate hydrolase family protein; PK, protein kinase; PNP, purine nucleoside phosphorylase; PSP, protein serine phosphatase; Rab_u, unclassified Rab; βTub, β-tubulin

The interactome, which was constructed based on all coIP experiments strongly supported interaction between TvMmm2b and TvMdm12 (Fig. 6, Additional file 4: Table S3). These proteins were reciprocally co-immunoprecipitated, resulting in 20 shared interacting proteins (10 are shown in Fig. 6). The shared proteins included two proteins with EF hand-type calcium-binding domains (TVAG_290210, TVAG_454360); additional three more EF-hand proteins co-immunoprecipitated with TvMdm12 (TVAG_378020, TVAG_157510, TVAG_037530), and one with TvMmm2b only (TVAG_454360) (Additional file 4: Table S3). Other TvMmm2b-TvMdm12 shared proteins included two paralogs of myeloid leukemia factors (TVAG_020600 and TVAG_150300), Rab22, a chaperon DnaJ-3 (TVAG_347420), a putative protein serine phosphatase (TVAG_274690), a protein with protein kinase domain (TVAG_452010), and two conserved hypothetical proteins (CHP-1, TVAG_373260; and CHP-2, TVAG_100110).

**Fig. 7:**
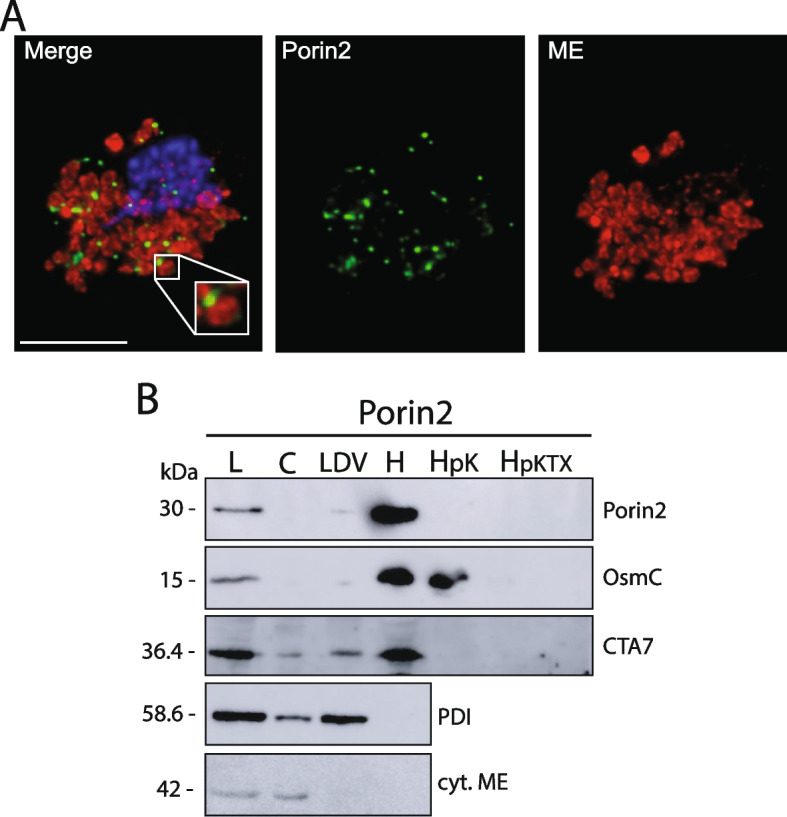
Cellular localization of *T. vaginalis* Porin2. **A** Immunofluorescent microscopy. Hemagglutinin (HA)-tagged recombinant Porin2 and hydrogenosomal marker malic enzyme (ME) were visualized using mouse α-HA (green) and rabbit α-malic enzyme (red) antibodies, respectively. The nucleus was stained with DAPI (blue). **B** Western blot analysis of cellular fractions isolated from the lysate of HA-tagged Porin2 expressing *T. vaginalis* cells. Cellular fractions were separated by differential and Percoll gradient centrifugation. L, cell lysate; C, cytosolic fraction; LDV, low-density vesicles; H, hydrogenosomal fraction. Hydrogenosomal fraction was treated with proteinase K (HpK) or proteinase K with Triton X-100 (HpKTX). Porin2, OsmC (hydrogenosomal marker), C-tail anchored protein 7 (CTA7), PDI (ER marker), and cytosolic malic enzyme (cyt. ME, cytosolic marker) were detected by mouse α-HA, rat polyclonal α-OsmC, rat polyclonal α-CTA7, rat polyclonal α-PDI, and mouse α-cyt. ME, respectively

Three baits, TvMmm2a, TvMmm2b, and TvMdm12, immunoprecipitated with a β-barrel protein Porin2 (TVAG_340380). As we did not identify Mdm10 in the *T. vaginalis* genome, we hypothesized that Porin2 may serve as a functional analog of Mdm10. Homology searches across organisms with hydrogenosomes identified Porin2 in all parabasalids. Phylogenetic analysis revealed that Porin2 forms a well-supported cluster, which is distinct from Mdm10 and other mitochondrial beta-barrel proteins (Tom40 and Porin, Additional file 2: Fig. S5). The branching order suggested that Porin2 might be an extremely divergent Mdm10. However, the low statistical support did not allow an unambiguous interpretation of the Porin, Porin2, and Mdm10 relationship. Noteworthy, Porin2 is absent in other organisms including related *A. flamelloides* that possess Mdm10 (Additional file 3: Table S2, Additional file 2: Fig. S5). To verify Porin2 localization, its HA-tagged version was expressed in *T. vaginalis*. Porin2 appears as dots, mostly associated with the periphery of hydrogenosomes (Fig. 7A). The hydrogenosomal localization of Porin2 was further supported by western blot analysis of subcellular fractions (Fig. 7B). Proteinase K treatment of isolated hydrogenosomes revealed that the signal for Porin2 was not membrane-protected, which indicated that HA-tag epitope was exposed to the cytosol as in the control CTA7, while matrix protein OsmC was not affected (Fig. 7B).

Next, we used Porin2 as a bait, which revealed 35 coIP proteins (Additional file 4: Table S3). This dataset includes a mitochondrial carrier protein (TVAG_197670) that was common with TvMmm2b and TvMdm12 coIP datasets. The ERMES components were not pulled down except TvMdm12; however, its enrichment was under the threshold of significance. Interestingly, the Porin2 coIP with four other β-barrel proteins, Hmp36-1, Hmp36-2, Hmp35-2, and Sam50. In addition, it shares a PloopNTPase (TVAG_190360) with TvMmm1b bait, and ten additional paralogs of PloopNTPases coIP with Porin2 only (Fig. 6, Additional file 4: Table S3).

CoIP experiments further supported possible interactions between TvMmm1a and TvMmm1b (Fig. 6). TvMmm1a pulled down TvMmm1b, and both TvMmm1 paralogs shared three proteins including Mmm1d protein (TVAG_194830). TvMmm1a shares three other proteins with TvMdm12 (Fig. 6).

Interestingly, multiple ERMES components (3–4) pulled down chaperones including Hsp70-1 (TVAG_291920), Hsp70-2 (TVAG_479220), Hsp70-3 (TVAG_107710), Hsp70-4 (TVAG_163000), co-chaperone DnaJ-1 (class I Hsp40 family, TVAG_305730), and DnaJ-2 (class II Hsp40 family, TVAG_317210) (Fig. 6). Phylogenetic reconstruction of *T. vaginalis* Hsp70 paralogs (41 sequences) revealed that Hsp70-1, Hsp70-2, and Hsp70-3 formed a distinct well-supported cluster (Additional file 2: Fig. S6). These Hsp70 proteins are fused at C-terminus with domains of 398–406 amino acid residues without known homology, which are not present in other Hsp70 paralogs.

### Modeling of ERMES complex indicates the formation of the hydrophobic tunnel between ER and hydrogenosomes

**Fig. 8:**
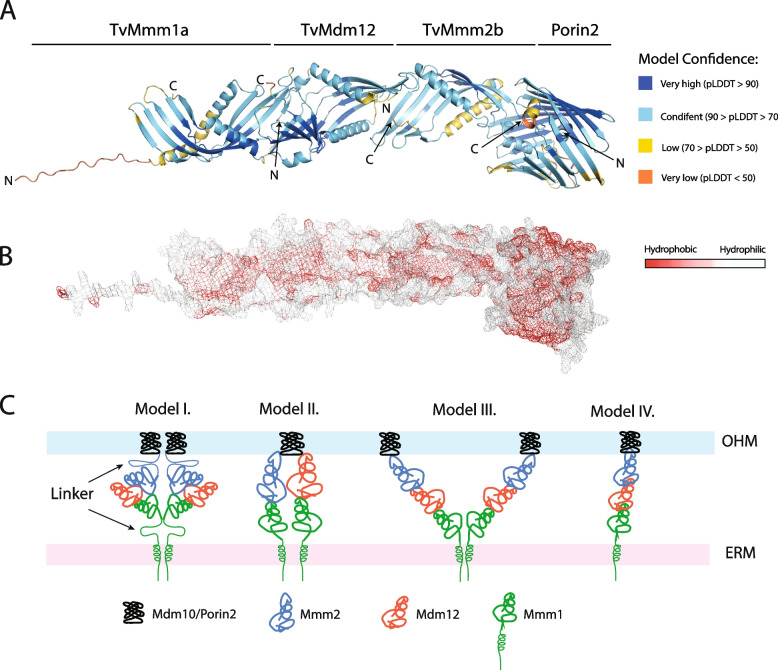
Structural model of ERMES complex in *T. vaginalis.* **A** Model of the ERMES Tvmm1a-TvMdm12-TvMmm2b-Porin2 heterotetramer as predicted by ColabFold. Predicted domains are colored according to the pLDDT score of confidence. Arrows pointed at C-(carboxy) and N- (amino) terminus of each subunit. **B** Visualization of the hydrophobic amino acid residues in the inner surface of the ERMES conduit. The ERMES complex is shown as mesh and each amino acid is colored by its hydrophobicity score as defined by the color_h.py script. **C** Models of possible arrangements of ERMES components tethering ER and mitochondrial (hydrogenosomal) membranes. Model I. The shuttle model, Mmm1 and Mmm2 flexible linkers allow the complex movement between membranes [2]. Model II. In the two-tunnel model, Mmm1 is dimerized and each Mmm1 subunit interacts with a different partner (Mdm12, Mmm2) [32]. Model III. Mmm1 is dimerized and each subunit forms a continuous conduit with Mdm12-Mmm2 that is anchored to mitochondrial/hydrogenosomal membrane via Mdm10/Porin2, respectively ( [3], this work). Model IV. A single continuous conduit was reconstructed from light and electron cryo-microscopy [33]

To predict structural protein–protein interactions between *T. vaginalis* ERMES subunits and the architecture of the heterodimeric complex, we used ColabFold software based on MMseqs2 homology searches [31]. As yeast Mmm1 forms homodimers in vitro, first we tested TvMmm1 paralogs for the homodimer modeling (Additional file 5: Table S4). When the complete TvMmm1a sequence was used, pLDDT metric value was < 60 (pDockQ = 0.112) indicating low model confidence; however, the removal of N-terminal TMD, which is embedded in ER membrane (1–33 AA), resulted in the well-supported model (pLDDT > 76, pDockQ = 0.232). The symmetric dimer revealed a head-to-head arrangement of TvMmm1a subunits with the interface composed of two α1-helices and β1-4 strands (Additional file 2: Fig. S7). In contrast, the modeling of TvMmm1b and TvMmm1c did not support the formation of homodimers regardless of TMD presence or absence (Additional file 5: Table S4). Modeling of TvMmm1d and TvMmm1e predicted the formation of homodimers with good accuracy (pLDDT > 70, pDockQ > 0.23, Additional file 5: Table S4). Next, we modeled heterodimeric combinations that supported the formation of a tubular structure with pLDDT ranging from 70 to 81 (pDockQ > 0.23, Additional file 5: Table S4). The modeling of the TvMmm1a-TvMdm12 complex revealed the head-to-tail structure of the heterodimer, which resembles the previously reported crystal structure of yeast Mmm1-Mdm12 dimer [3] (Additional file 2: Fig. S8). N-terminus of TvMdm12 (referred to as the head) including α1 and α2-helix, β1-strand, and the loop preceding the antiparallel β2-strand (residues YELPQLQQIPFNAP) form an interface with the distal end of TvMmm1a (referred to as the tail) that includes TvMmm1a β3-strand and its preceding loop (residues GPIDIPQL), β4-strand, the following loop (residues LLDDPKNASQKHI), and α3-helix (Additional file 2: Fig. S8). Multiple hydrophobic interactions and H-bonds were predicted within the interface (Additional file 2: Fig. S8). The yeast Mmm1-Mdm12 subunits form in vitro a crescent-shaped heterotetramer with a central Mmm1 dimer, which is flanked by two Mdm12 molecules [2]. The modeling of the TvMmm1a-TvMdm12 tetramer resulted in the formation of a comparable structure with pLDTT 77–79 (Additional file 5: Table S4). However, the superposition of both complexes predicted a considerably smaller angle (~ 102° yeast versus ~ 141.5° *T. vaginalis*) between the axes of two tubular structures of the model and a displacement of the second tubular structure by ~ 57° (Additional file 2: Fig. S9). Next, we focused on modeling TvMmm2b and TvMdm12 heterodimer complex because interactions between these two subunits were strongly indicated by coIP data. The model predicted the formation of a TvMmm2b-TvMdm12 head-to-tail tubular structure. The interface between SMP domains was formed by TvMmm2b N-terminal loop, α1-helix, which lies adjacent to the proximal TvMdm12 loop (residues DKIAPG) and TvMdm12 β2- and β3-strand, and TvMmm2b β1-strand, which interacts with TvMdm12 β4-strand and α4-helix and with the loop between them (residues GAEEKVFD) (Additional file 2: Fig. S10). Based on coIP data, we hypothesized that the interaction between TvMmm2b and Porin2 may serve as an anchor for the ERMES complex. Indeed, the modeling predicted that TvMmm2b is associated with the Porin2 via the α2-helix that buries within the Porin2 channel with pLDDT support 81 and 77 for TvMmm2b and Porin2, respectively (pDockQ = 0.232, Additional file 5: Table S4, Additional file 2: Fig. S11). As a negative control, we tested a complex formation between TvMmm2b and another β-barrel protein Tom40, which was not identified in the interactome. The TvMmm2b-Tom40 model revealed low pLDDT support for heterodimer formation (pDockQ = 0.047 and pLDDT 59 and 50, respectively). Finally, we modeled the structure of TvMmm1a, TvMdm12, TvMmm2b, and Porin2 heterotetramer. This modeling showed the formation of a continuous tubular-shaped tunnel of TvMmm1-TvMdm12-TvMmm2b with the length ~ 140 Å, which was anchored to Porin2 channel (Fig. 8A). The surface of the tubular structure was predicted to be predominantly hydrophobic (Fig. 8B), which is consistent with the involvement of ERMES in phospholipid trafficking.

### Distribution of Psd1 and CLST

ERMES is involved in the import of phosphatidylserine (PS) and cytidine diphosphate diacylglycerol (CDP-DAG) from ER into mitochondria where they are further metabolized [34]. PS is converted to phosphatidylethanolamine (PE) by Psd1, and CDP-DAG is used for cardiolipin synthesis, with the key enzyme cardiolipin synthase (CLS). Thus, we were interested in whether these pathways operate in *T. vaginalis* and other organisms with ERMES and hydrogenosomes (Fig. 2, Additional file 3: Table S2). No Psd1 candidates were identified in Parabasalia and other metamonads except for *A. flamelloides*, which possesses Psd1 with predicted N-terminal mitochondrial targeting signals (NTS). Moreover, Psd1 was found in *Pygsuia biforma* and anaerobic fungi (Fig. 2, Additional file 3: Table S2).

**Fig. 9:**
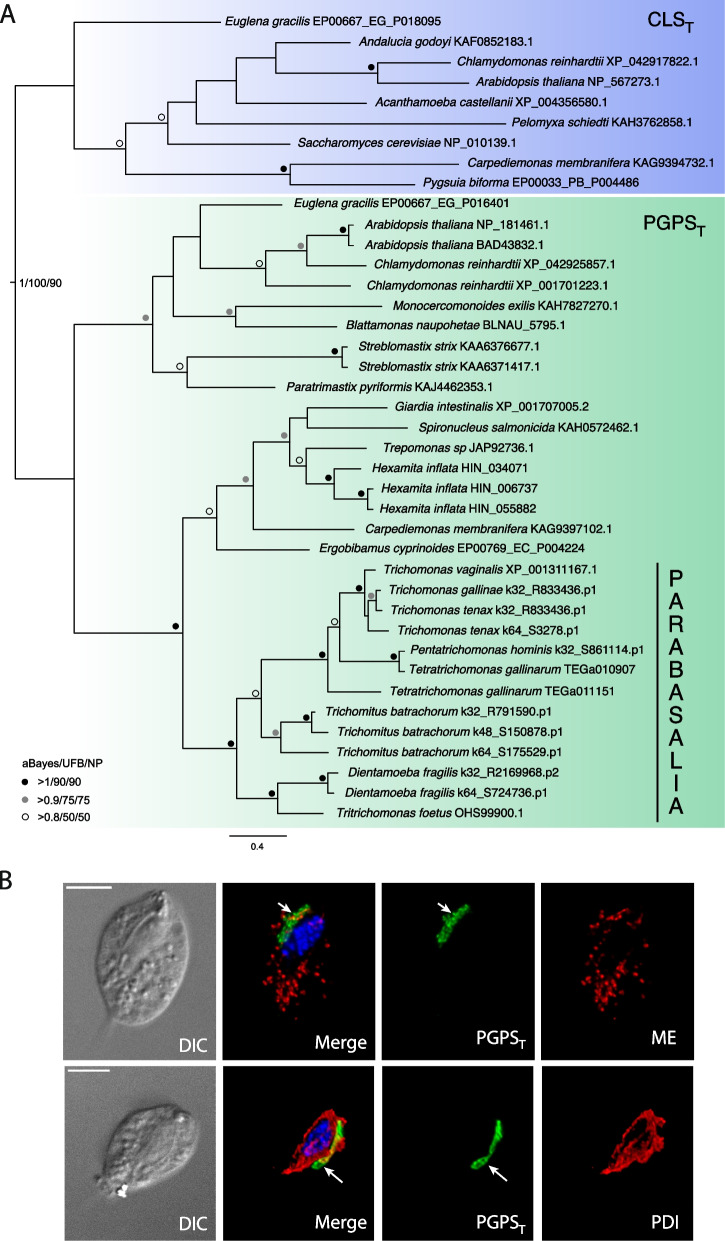
*T. vaginalis* possesses phosphatidylglycerol phosphate synthase (PGPS_T_) but not cardiolipin synthase (CLS_T_). **A** Phylogenetic analysis of PGPS_T_ and CLS_T_. The maximum likelihood (ML) tree was constructed using IQ-TREE (Best fit; LG + F + I + G4 model) with 40 sequences and 186 sites. Bootstrap support values and aBayes posterior probability were calculated using 1000 replicates each. The support values are represented in the order of aBayes (posterior probability value)/ML ultra-fast bootstrapping)/ML Non-parametric bootstrapping. **B** Cellular localization of *T. vaginalis* PGPS_T_. Hemagglutinin (HA)-tagged PGPS_T_ was expressed in *T. vaginalis* and visualized using mouse α-HA antibody (green). The malic enzyme (ME), a hydrogenosomal marker, was visualized with rabbit α-ME enzyme (red) antibody. Alternatively, HA-tagged PGPS_T_ was co-expressed with V5-tagged protein disulfide isomerase (PDI), an ER marker that was detected with rabbit α-V5 (red) antibody. The nucleus was stained with DAPI (blue). DIC, differential interference contrast. Bar = 5 μm

HMMER searches for eukaryotic CLS with transferase mechanism (CLS_T_) [29] identified homologous proteins in *T. vaginalis* and other metamonads (Additional file 3: Table S2). However, phylogenetic analysis revealed that metamonad sequences cluster together with phosphatidylglycerol phosphate synthase (PGPS) with a transferase mechanism (PGPS_T_, EC 2.7.8.5) (Fig. 9A) [29]. These sequences do not possess NTS and were predicted to target ER (Additional file 3: Table S2). CLS_T_ sequences formed a separate well-supported clade that includes two anaerobic protists *C. membranifera* and *Pygsuia biforma* (Fig. 9A).

To verify cell localization of *T. vaginalis* PGPS_T_ (TVAG_010140), we derived *T. vaginalis* cell line expressing PGPS_T_ with a C-terminal hemagglutinin (HA) tag and prepared double transfectants expressing HA-tagged PGPS_T_ and V5-tagged PDI. Confocal immunofluorescence microscopy revealed that PGPS_T_ localized into the perinuclear rod-like structure, which was distinct from ER and corresponded to the morphology of the Golgi complex (Fig. 9B). No PGPS_T_ signal colocalized with the hydrogenosomal malic enzyme, which argues against its function as CLS.

These data indicate that hydrogenosomal phospholipid metabolism was lost in multiple lineages with hydrogenosomes including *T. vaginalis*, most other metamonads, and Archamoebae. However, the finding of Psd1 in *A. flamelloides* and CLS in *C. membranifera* indicates that hydrogenosomal phospholipid metabolism was present in a common ancestor of Metamonada.

### TvMdm12 interacts with phosphoinositides

**Fig. 10:**
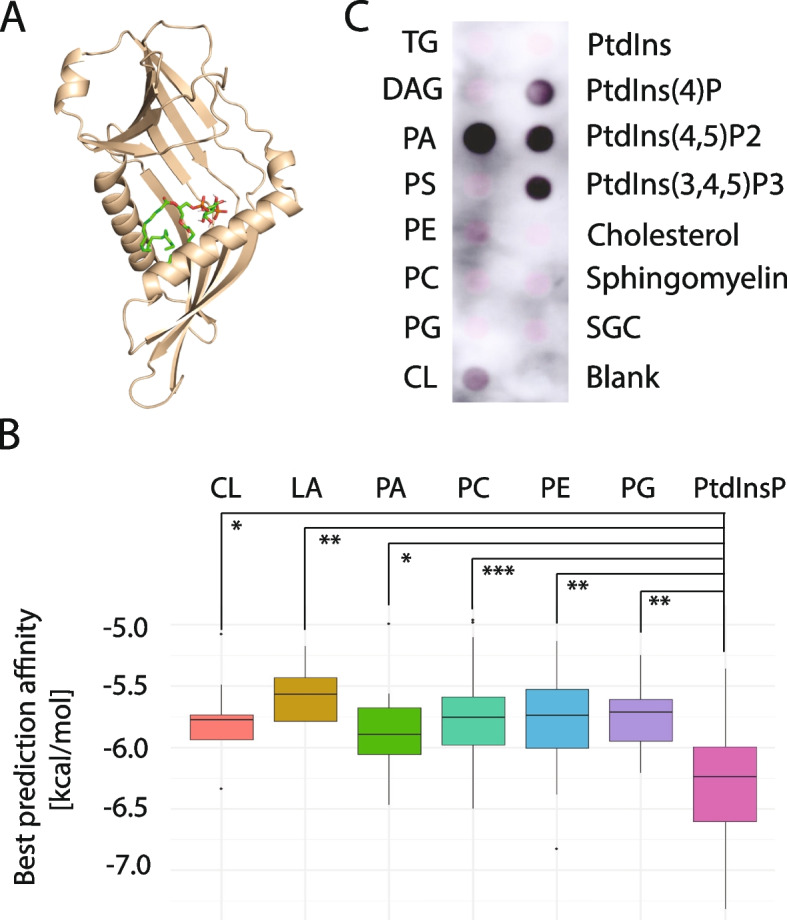
Interactions of *T. vaginalis* Mdm12 with phospholipids. **A** Example of docking of phosphatidylinositol 4-phosphate (PBD ID T7M) into TvMdm12 as predicted by AutoDock Vina v1.2.3 [36]. Green, red, orange, and white represent carbon, oxygen, phosphate, and hydrogen, respectively. **B** Boxplot of ligand binding affinities. Individual colored boxes illustrate groups of phospholipids. The single box represents Inter quartal range (IQR) with a horizontal line showing the median. Whiskers represent maximum (Q3 + 1.5*IQR) and minimum (Q1 − 1.5*IQR) values. Dots indicate outliers. The significance of the difference among the groups was assessed by ANOVA (*F-*value = 4.56, *P*-value = 0.0003, Degree of freedom = 6) and followed by a post hoc test (Tukey). Stars indicate the significance of the group versus PIP (*p*-value< 0.05 = *, *p*-value< 0.01 = **, *p*-value 0.001 = ***). CL, cardiolipin; PA, phosphatidic acid; PC, phosphatidylcholine; PE, phosphatidylethanolamine; LA, lysophosphatidic acid; PG, phosphatidylglycerol; PtdInsP, phosphatidylinositolphosphate. **C** Binding of recombinant *T. vaginalis* TvMdm12 to lipids by protein-lipid overlay assay. Membrane lipid strips were incubated with recombinant TvMdm12. The binding of TvMdm12 to immobilized lipids in the spots was detected by mouse monoclonal α-His antibody and α-mouse antibody conjugated with horseradish peroxidase. TG, triglyceride; DAG, diacylglycerol; PA, phosphatidic acid; PS, phosphatidylserine; PE, phosphatidylethanolamine; PC, phosphatidylcholine; PG, phosphatidylglycerol; CL, cardiolipin; PtdIns, phosphatidylinositol; PtdIns(4)P, phosphatidylinositol 4-phosphate; PtdIns(4,5)P2, phosphatidylinositol 4,5-bisphosphate; PtdIns(3,4,5)P3, phosphatidylinositol 3,4,5-trisphosphate; SGC, 3-sulfogalactosylceramide

To investigate the nature of phospholipids that are transported via ERMES, we selected the TvMdm12 subunit and tested the ability of its SMP domain to bind phospholipids using AutoDock Vina software for molecular docking. TvMdm12 structure within the TvMmm1-TvMdm12-TvMmm2b model (Fig. 8) was used for the docking of 128 ligands of seven phospholipid categories (Fig. 10, Additional file 6: Table S5). The best prediction affinity was found for phosphatidylinositol phosphates (PtdInsP) with the median of the best scores for 18 PtdInsP structures − 6.2 kcal/mol. The second best category was phosphatidic acids (PA, − 5.8 kcal/mol) (Fig. 10A, and B, Additional file 6: Table S5). Next, we tested recombinant TvMdm12 for ligand binding specificity using protein-lipid binding assay (Fig. 10C). TvMdm12 bound to PtdIns 4-phosphate, PtdIns 4,5-bisphosphate, PtdIns 3,4,5-trisphosphate, and phosphatidic acid. Low interaction was also observed with cardiolipin, although cardiolipin is likely absent in vivo [35]. In combination with the results from molecular docking, PtdInsPs appeared to be the best candidates to be transported by ERMES in *T. vaginalis*.

## Discussion

Hydrogenosomes evolved independently in multiple eukaryotic lineages as an adaptation to an anaerobic lifestyle. They lost a majority of mitochondrial pathways including oxidative phosphorylation and gained enzymes for anaerobic breakdown of pyruvate linked to ATP synthesis via substrate-level phosphorylation. The presence of ATP synthesis distinguishes hydrogenosomes from mitosomes that entirely lost energy metabolism [37]. The ERMES complex was hypothesized to be another differentiating feature between these organelles being retained in hydrogenosomes and lost in mitosomes [22]. Indeed, our cell localization studies, proteomic analysis-based interactome, and structural predictions provided the first experimental evidence that the ERMES complex operates in model parabasalid, *T. vaginalis*. As a similar set of ERMES orthologs is present in other members of the Parabasalia group, we can infer that the presence of ERMES is a common feature of this lineage. However, our searches across other eukaryotic lineages with hydrogenosomes revealed that the presence of the ERMES complex is not a common hydrogenosomal feature. Most metamonads with hydrogenosomes, including diplomonads and retortamonads, are devoid of ERMES. The genes coding the ERMES complex proteins are retained only in free-living basal fornicate *C. membranifera*, and we found a gradual loss of ERMES components in other free-living carpediemonas-like organisms [38]. Archamoebae is another group of anaerobes that lost ERMES in species with hydrogenosomes (*M. balamuthi* and *P. schiedti*), although it is present in Mycetozoa, a related lineage with mitochondria [39]. This distribution indicates that ERMES was lost in the very early steps of reductive evolution in Fornicata and Archamoebae and that ERMES losses were not directly associated with losses of energy metabolism in organisms with mitosomes.

The major difference in ERMES subunit composition between *T. vaginalis* and yeast is the anchoring subunit of the complex to the outer hydrogenosomal membrane. Our results indicate that in trichomonad hydrogenosomes, Mdm10 is replaced by beta-barrel protein Porin2, which is known to be an integral protein of the hydrogenosomal membrane, however, its function was unclear [40]. We showed that Porin2 coIP with TvMdm12, TvMmm2a, and TvMmm2b, but not with TvMmm1 proteins. The coIP data are consistent with confocal microscopy that revealed an association of TvMdm12 and both TvMmm2 paralogs with hydrogenosomes, while TvMmm1 paralogs localized in *T. vaginalis* ER. The failure of reciprocal coIP of ERMES subunits using Porin2 as bait suggests a weak or transient nature of ERMES complex assembly as observed previously [2, 41]. The HMM searches identified Porin2 orthologs in all parabasalids while Mdm10 was absent. Interestingly, inverse distribution was found in *A. flamelloides* of Anaeramoebae group that lacks Porin2 but possesses Mdm10. This group has been shown to be a sister of the Parabasalia and to have retained more canonical mitochondrial features [26]. Indeed, we found that *A. flamelloides* possesses Psd1, an enzyme of mitochondrial phospholipid metabolism converting PS to PE. In yeast, ERMES was proposed to facilitate an import of Psd1 substrate (PS) from ER to mitochondria and an export of the product (PE) backward to ER [1]. Thus, the presence of Mdm10 and Psd1 in *A. flamelloides* suggests that this organism contains a rather standard ERMES structure linked with phospholipid metabolism and bidirectional transport of metabolites. In contrast, parabasalids lost the hydrogenosomal Psd1, and thus, the function of ERMES is most likely reduced to the unidirectional delivery of phospholipids to the hydrogenosomal membranes. Whether the replacement of the Mdm10 with Porin2 in Parabasalia is related to the changes in ERMES function remains to be established. However, a strong phenotypic correlation between the function of Psd1 and ERMES subunits has been reported [1].

Cardiolipin synthesis is another mitochondrial pathway that is possibly dependent on ERMES-mediated transport of the glycerophospholipid substrate (PA), which efficiently binds to yeast Mmm1-Mdm12 complex [3]. In *T. vaginalis*, the presence of cardiolipin is a controversial issue. A phospholipid with cardiolipin characteristics has been detected in isolated hydrogenosomes of *Tritrichomonas foetus* [42, 43], while other studies of *T. foetus* and *T. vaginalis* reported the absence of cardiolipin [35, 44]. Curiously, CLS was currently annotated in the genome of *T. vaginalis* reference strain (TVAGG3_0082770, TrichDB). This gene is identical to the one with accession number TVAG_010140, which we identified as putative PGPS_T_. CLS_T_ and PGPS_T_ share a common origin, which may lead to confusion [29]. We showed that PGPS_T_ proteins including *T. vaginalis* ortholog form a distinct well-supported clade, while a different topology was observed for CLS_T_. Moreover, while CLS_T_ is located exclusively in mitochondria, PGPS_T_ is present primarily in the ER [45]. We found that none of *T. vaginalis* PGPS_T_ paralogs possesses hydrogenosomal targeting sequences, they were not identified in any hydrogenosomal proteome [40, 46, 47], and PGPS_T_ (TVAG_010140) did not localize to *T. vaginalis* hydrogenosomes (this study). *T. vaginalis* PGPS_T_ seems to localize to Golgi, which is unexpected, but more consistent with the function of PGPS_T_ than CLS_T_. These analyses indicate the absence of CLS in *T. vaginalis*, which is in agreement with previous reports on no detectable cardiolipin in this organism [35, 44].

Another unusual feature of parabasalid ERMES components is the presence of multiple Mmm1 copies which are not identical in their structure and possibly function. In *T. vaginalis*, three Mmm1 paralogs (TvMmm1a, b, and c) contain N-terminal TMD, as archetypal fungal Mmm1, to be anchored in ER membrane, while two paralogs lack this anchor (TvMmm1d and TvMmm1e). What is the function of TvMmm1d and TvMmm1e is not clear. In yeast, Mmm1a forms a homodimer anchoring the ERMES complex to the ER membrane [3]. Our structural modeling suggested that TvMmm1d can form a heterodimer with TvMmm1a, which was further supported by coIP experiments. Such a complex might be anchored to ER via TvMmm1a and associated with other ERMES components. Alternatively, the structural modeling predicted the formation of TvMmm1d and TvMmm1e homodimers. As these proteins possess a lipid-binding SMP domain but lack TMD, they may serve as ERMES-independent soluble lipid transporters [48].

ERMES is the best-studied tether between ER and mitochondria, although its overall structure is still under investigation. Several models for ERMES complex assembly and lipid transport have been proposed. According to the shuttle model (Model I, Fig. 8C), Mmm1/Mdm12/Mmm2 complex transports phospholipids back and forth between ER and mitochondrial membranes with the exchange of lipids between subunits via lateral opening along the SMP domain [2]. The movement of the complex between membranes is mediated by long flexible linkers at the N-terminus of Mmm1 and the C-terminus of Mmm2. However, we found that *T. vaginalis* TvMmm1 and TvMmm2 lack these flexible linkers; thus, the shuttle model is unlikely to explain the mode of ERMES operation. The tunnel model proposed the formation of a continuous conduit with an outlet and inlet through which the lipid diffuses. As Mmm1 was shown to form a homodimer [3], the assembly of two channels has been proposed, in which one Mmm1 subunit interacts with Mdm12 and the second with Mmm2 (Model II) [32], or the two channels may each consist of Mmm1/Mdm12/Mmm2 subunits (Model III) [49]. Our model of *T. vaginalis* ERMES predicted a possibility that TvMmm1a-TvMdm12-TvMmm2b form a continuous hydrophobic channel that is anchored to ER via N-terminal TMD of TvMmm1a, and to outer hydrogenosomal membrane via Porin2 (Model III). Similarly to yeast [3], TvMmm1a was predicted to form a head-to-head oriented homodimer of a V-shape, though with a smaller angle between the axes of two arms in comparison to the yeast complex. TvMmm1a-TvMdm12 was predicted to interact in a tail-to-head manner in agreement with the yeast Mmm1-Mdm12 complex [3]. In addition, we predicted a similar tail-to-head arrangement between TvMdm12 and TvMmm2b. Moreover, our model suggested that TvMmm2b may associate with Porin2 via the alpha-helix that is buried within the beta-barrel cavity. During the preparation of this manuscript, a similar structure of *S. cerevisiae* ERMES has been reported [33]. The in silico AlphaFold-based tools suggested linear conformation of the yeast Mmm1-Mdm12-Mmm2 heterotrimer as we observed for *T. vaginalis* components. Moreover, the formation of a continuous string with the same subunit order of yeast ERMES subunits was supported by cryo-correlative microscopy [33]. Interestingly*,* the majority of in situ observed yeast complexes revealed a single bridge arrangement without the formation of Mmm1 homodimers, (Model IV, Fig. 8). Whether TvMmm1 forms a dimeric or monomeric structure in situ is currently unknown; however, the presence of multiple TvMmm1 paralogs with different predicted capability to form homodimers suggests both possibilities.

The function of ERMES in *T. vaginalis* and other organisms with hydrogenosomes remains to be established. Nevertheless, observed differences in ERMES composition and hydrogenosomal phospholipid metabolism imply the evolution of lineage-specific variability. In *T. vaginalis*, our experiments suggested that ERMES might be involved in the transport of PtdInsPs. This group of phospholipids ranked the best in molecular docking into the SMP cavity of TvMdm12 (the central ERMES subunit) and the recombinant TvMdm12 bound to PtdInsPs in the protein-lipid binding assay. *S. cerevisiae* Mdm12 was shown to bind preferentially phosphatidylcholine (PC), which is synthesized in ER, and PE and phosphatidylglycerol (PG), which are synthesized in the mitochondria [2, 34]. However, *T. vaginalis* lacks enzymes for PE and PG synthesis in hydrogenosomes, as well as de novo synthesis of PC [50]. Therefore, different ERMES affinities could be expected in *T. vaginalis*. PtdInsPs are signaling lipids that are present in all eukaryotic membranes including mitochondria [51, 52]. They are synthesized by phosphatidylinositol synthase (Pis) that predominantly localizes to ER. Two paralogs of Pis are annotated in *T. vaginalis* genome (TrichDB), which supports the possibility that PtdInsPs are available in ER for their transport from ER via ERMES to hydrogenosomal membranes. Nevertheless, the specificity of TvMdm12 for PtdInsPs and their role need to be further studied.

In addition to lipid transport, MCSs are critical for ER-mitochondria calcium transport [9, 53]. In hydrogenosomes, calcium has been shown to accumulate in intermembrane peripheral vesicles, and some of these vesicles were observed in close proximity to the ER membrane [54]. Our coIP experiments pointed to six proteins with calcium-binding EF-hand domains that coIP with ERMES components. These proteins of a diverse range of functions may serve as calcium sensors, calcium signal modulators, or participate in calcium homeostasis [55]. Although the function of EF-hand proteins that coIP with *T. vaginalis* ERMES is unknown, they represent interesting candidates to study the ER-hydrogenosome-calcium-ERMES interplay.

## Conclusions

We demonstrated that the divergent ERMES complex with a specific Porin2 subunit is conserved in protists with hydrogenosomes of the Parabasalia group, and we utilized *T. vaginalis* as a model parabasalid organism to provide initial evidence of parabasalid ERMES cell localization, function, and structure. However, we also demonstrated that ERMES was reduced or lost in protists of Fornicata and Archamoebae lineages regardless if they possess ATP-generating hydrogenosomes or energetically silenced mitosomes.

These findings open multiple questions such as the following: (i) What was the evolutionary pressure to retain ERMES in Parabasalia? The ERMES is functionally linked with ER-mitochondrion (hydrogenosome) phospholipid metabolism. However, in Parabasalids, the metabolism of phospholipids is very limited in ER and lost in hydrogenosomes. Therefore, it is tempting to speculate that ERMES acquire either novel functions such as the transport of PtdInsPs as possibly suggested by our experiments, or it is involved in other physiologically important functions such as the maintenance of calcium homeostasis. (ii) When ERMES is lost, how phospholipids are delivered into the hydrogenosomal/mitosomal membrane? The losses imply that other lipid transport mechanisms replaced the function of ERMES. In aerobic eukaryotes that lack ERMES, several alternative systems were proposed to substitute the ERMES function including vCLAMP, Vps13, and Lamp6 [22]. Whether these systems mediate ER-hydrogenosome interactions in metamonads and Archamoebae remains to be clarified. Nevertheless, Lamp6 interact with mitochondrial Tom70 subunits of TOM complex at MOM, respectively, which are absent in Archamoebae and metamonads; thus, its involvement in lipid transport is unlikely in these lineages [56]. Most anaerobic protists with hydrogenosomes and all with mitosomes are parasitic or endobiotic species, which are unable to synthesize the majority of phospholipids. Consequently, phospholipids are taken up from their environment [57]. However, it is virtually unknown how the acquired phospholipids are sorted within these parasites and targeted to the membranes of their organelles. Elucidation of these questions will contribute not only to a better understanding of ERMES evolution in anaerobic protists but more importantly to the evolution of parasitism.

## Methods

### Cell cultivation

*T. vaginalis* strain T1 [58] was cultivated at 37 °C in Diamond’s Trypton-Yeast extract-Maltose (TYM) medium, supplemented with 10% inactivated horse serum, and adjusted to pH 6.2 [59]. Single transformants were selected in TYM with 200 μg/ml of geneticin (G418), and double transformants were selected using 200 μg/ml G418 and 40 μg/ml puromycin as described [60].

### Gene cloning and tagged protein expression

The genes encoding TvMmm1a (TVAG_214860), TvMmm1b (TVAG_302900), TvMmm2a (TVAG_217400), TvMmm2b (TVAG_375920), TvMdm12 (TVAG_063000), Porin2 (TVAG_340380), and PGPS_T_ (TVAG_010140) were amplified from *T. vaginalis* genomic DNA by PCR (Additional file 7: Table S6) and cloned into the vector TagVag-HA-Neo for protein expression with di-hemagglutinin (HA) tag at the C-terminus in *T. vaginalis* under G418 selection. Protein disulfide-isomerase (PDI, TVAG_267400) and TvMmm2b (TVAG_375920) were expressed with a C-terminal 3xV5 tag using the vector pTagVag-V5-Pur under puromycin selection [60]. The cells were transformed using electroporation as described [61].

### Subcellular fractionation and protease protection assay

*T. vaginalis* logarithmic culture (500 ml) was harvested by centrifugation and homogenized by sonication. Subcellular fractions (cytoplasm, vesicular fraction, and hydrogenosomes) were isolated by differential and Percoll gradient centrifugation as described [62].

A protein protection assay was performed using proteinase K according to [30]. Samples were analyzed by immunoblotting using mouse monoclonal anti-HA antibody (Ab) (ExBio, Vestec, Czech Republic), rat polyclonal anti-TvOsmC Ab [63], and anti-CTA7 Ab [30]. Proteins were visualized by secondary anti-mouse, anti-rat, or anti-rabbit Abs fused with horseradish peroxidase using chemiluminescence (Immobilon Classico Western HRP substrate, Millipore). Raw blots are available in Additional File 2: Fig. S12-S15.

### Immunofluorescence microscopy

*T. vaginalis* cells were fixed in 2% formaldehyde and processed as described [30]. HA-tagged and V5-tagged proteins were detected by mouse monoclonal anti-HA Ab (ExBio, Vestec, Czech Republic) and rabbit monoclonal anti-V5 Ab (Abcam, Cambridge, United Kingdom), respectively. Hydrogenosomal malic enzyme was detected with rabbit polyclonal anti-malic enzyme Ab [64]. Secondary antibodies were Alexa Fluor 488 donkey anti-mouse Ab and Alexa Fluor 594 donkey anti-rabbit Ab (Thermo Fisher). Slides were observed with Zeiss Elyra PS.1 microscope using Structured Illumination Microscopy (SIM), and confocal microscope Leica TCS SP8 WLL SMD-FLIM. Acquired images were deconvolved using Huygens 19.04 software (Scientific Volume Imaging) and processed with the Imaris 9.7.2 Package for Cell Biologists (Bitplane AG, Zurich, Switzerland). Voxel-based co-localization was performed using ImarisColoc and Pearson correlation coefficient (PCC) in co-localized volume was calculated as described [65].

### Co-immunoprecipitation (coIP) of protein complexes

CoIP was performed as described [56]. Briefly, *T. vaginalis* T1 strain and transfected cell lines expressing HA-tagged proteins (baits) were incubated with 1 mM of the crosslinker dithiobis(succinimidyl propionate) (DSP, Sigma-Aldrich, St. Louis, MO, USA) at room temperature for 30 min. The reaction was stopped with 50 mM tris(hydroxymethyl)aminomethane (TRIS), pH 7.5. Cells were washed with phosphate-buffered saline (PBS, pH 7) and solubilized in IP buffer (50 mM TRIS, 150 mM NaCl, pH 7.2) with 1% Triton X-100. The suspension was incubated with Dynabeads (Thermo Fisher Scientific, Waltham, Massachusetts, USA) coupled with anti-HA Ab (ExBio, Prague, Czech Republic) for 90 min on a rotator at room temperature. The beads were washed with IP buffer and proteins were eluted in 100 mM triethylammonium bicarbonate (TEAB) containing 1% sodium deoxycholate (Sigma-Aldrich, St. Louis, MO, USA). Three independent coIP experiments were performed for each bait.

### Label-free quantitative (LFQ) MS analysis

Eluted proteins were digested with trypsin and peptides were purified as described [66]. Peptides were separated by nano-scale liquid chromatography (UltiMate 3000 RSLC, Thermo Scientific) using reversed-phase column (EASY-Spray column, 50 cm × 75 μm ID, PepMap C18, 2 μm particles, 100 Å pore size) that was coupled with an Orbitrap Fusion Tribrid mass spectrometer (Thermo Scientific). Raw data were processed with the MaxQuant software (version 1.6.3.4) [67]. The precursor ion mass tolerance in the initial search was 20 ppm, the tolerance in the main search was 4.5 ppm, and the fragment ion mass tolerance was 0.5 Da. Genomic sequences and gene annotation were obtained from the database TrichDB (release 2020–05-27, 60.330 entries) [68]. Quantifications were performed with the label-free algorithm and data was evaluated using Perseus 1.6.2.3 software with a false discovery rate (FDR) for proteins 0.05, and S = 1. The protein interactions network was visualized with Cytoscape v3.9.1. [69]. The mass spectrometry proteomics data have been deposited to the ProteomeXchange Consortium via the PRIDE [70] partner repository with the dataset identifier PXD044071.

### Homology searching of ERMES components and lipid synthesis enzymes

Publicly available genomes, transcriptomes, and protein datasets were obtained from NCBI for *T. vaginalis* [71], *H. meleagridis* [72], and *T. foetus* [73]*.* For *T. gallinae*, *T. tenax*, *P. hominis*, *T. gallinarum*, *T. batrachorum*, and *D. fragilis* [74], transcriptomes were open-sourced at NCBI. The assembled transcriptome and protein dataset for *A. flamelloides* was acquired from the FigShare website https://doi.org/10.6084/m9.figshare.12205517.v1. [26]. Additionally for Pan-eukaryotic species, genomes, transcriptomes, and protein datasets were obtained from NCBI and EukProtv3 [75].

Initial homology searches of the ERMES components and lipid synthesis enzymes (phosphatidylethanolamine decarboxylase, Psd1; and cardiolipin synthase, CLS_T_) in the databases of parabasalids and *A. flamelloides* were conducted with *S. cerevisiae* queries using comparative genomics workflow AMOEBAE (Analysis of Molecular Evolution with Batch Entry) [27]. Forward searches were performed with BLASTp and tBLASTn with the *e*-value maximum limit of 0.05. The positive hits were then subjected to reciprocal/reverse BLAST in *S. cerevisiae* protein database, with identical parameters. Only positive hits from reverse searches were considered to be potential orthologues.

Based on the initial homology searches, Hidden Markov models built on *S. cerevisiae* and *T. vaginalis* protein sequences were used for HMMER searches in AMOEBAE to identify any previously unidentified homologs or to avoid any false-positive hits in Parabasalia and other lineages. Then, the clade-specific Hidden Markov models were built for a deep dive search into the specific lineages. All the positive hits were validated by searches for conserved functional domains using InterProScan [76], HHPred [77], and phmmer tool on HMMER web server [78]. TargetP2.0 [79], MitoFates1.2 [80], and DeepLOC2.0 [81] were used for protein cell localization predictions.

### Phylogenetic analysis

Protein sequences were aligned with MAFFT v7.505 [82]. Incomplete sequences were removed and the alignment was trimmed using Block Mapping and Gathering with Entropy (BMGE) [83]. Initially, the maximum likelihood tree was constructed using IQ-TREE v2.0 with ultrafast bootstrapping (-B -N 1000) [84]. ModelFinder was utilized to determine the best-scoring model for sequence evolution [85]. Based on long branching and low support values, alignments were adjusted and re-analyzed using IQ-TREE v2.0 with non-parametric bootstrapping and the Bayesian posterior probabilities values calculation (-b -N 1000 -alrt 1000 -abayes) [86].

### AlphaFold modeling and phospholipid docking

The structure of ERMES components and their interactions were predicted by the publicly available ColabFold notebook: AlphaFold2_mmseqs2 (https://github.com/sokrypton/ColabFold) [87]. The following parameters were used: model_type: alphafold2_multimer_v3; num_recycles: 12; and recycle_early_stop_tolerance: 0.5. The default options were used for the rest of the settings. Due to the size of the ERMES complex, two trimers were modeled (Mmm1a-Mdm12-Mmm2b and Mdm12-Mmm2b-Porin2). The final structure of ERMES was constructed by the superimposition of the two models in PYMOL v 2.5.2 (http://www.pymol.org/pymol). For further analysis and graphical outputs, three freely available scripts color_h.py, Interface Residues.py, and show_contacs.py were used for visualizing hydrophobic residues, highlighting the protein interaction surface, and the predicted protein contacts, respectively. Prediction scoring with pDockQ was calculated for each predicted dimer using the GitLab script (https://gitlab.com/ElofssonLab/FoldDock/-/tree/main) [88].

The phospholipid docking was performed using the Mdm12 subunit extracted from the ERMES tetramer by PyMOL v. 2.5.2. The ligands (128) for docking were downloaded from the Protein Data Bank (PDB) [89]; Mdm12 and the ligands were converted to pdbqt files using scripts from ADFR suite v. 1.0 [90] and Open Babel v. 3.1.1 [91], respectively. AutoDock Vina v. 1.2.3 was used to perform the docking [36]. Exhaustiveness was set to 20 and other parameters were kept on default. The results were visualized in PyMOL v. 2.5.2 and the statistical analysis and its visualization were performed in Rstudio with R v. 4.2.2. (www.R-project.org).

### Protein-lipid binding assay

His-tagged TvMdm12 were produced in *Escherichia coli* strain BL21 (DE3) using pET42B vector. The recombinant protein production was induced by 350 μM Isopropyl β-D-1-thiogalactopyranoside (IPTG) (Sigma-Aldrich, St. Louis, MO, USA) in LB medium and bacterial culture was grown at 37 °C for 4 h. Protein was isolated under native conditions on Nickel Agarose column (Sigma-Aldrich, St. Louis, MO, USA) in 50 mM NaH_2_PO_4_, 300 mM NaCl, pH 8 buffer as recommended by the manufacturer. Membrane Lipid Strip (P-6002, Echelon Biosciences Inc.) pre-spotted with 100 pmol of phospholipids (triglyceride (TG), diacylglycerol (DAG), phosphatidic acid (PA), phosphatidylserine (PS), phosphatidylethanolamine (PE), phosphatidylcholine (PC), phosphatidylglycerol (PG), cardolipin (CL), phosphatidylinositol (PtdIns), phosphatidylinositol 4-phosphate (PtdIns(4)P), phosphatidylinositol 4,5-bisphosphate (PtdIns(4,5)P2), phosphatidylinositol 3,4,5-trisphosphate (PtdIns(3,4,5)P3), cholesterol, sphingomyelin, 3-sulfogalactosylceramide (SGC), and blue blank) was blocked with 3% BSA and 0.1% Tween 20 in PBS (blocking buffer) overnight at 4 °C before incubation with recombinant His-tagged *T. vaginalis* Mdm12 (1 μg/ml) in blocking buffer for 1 h at room temperature (RT). After incubation, the strip has been washed three times with PBS containing 0.1% Tween 20 (PBS-T). Next, the strip has been incubated at RT with 1:1000 anti-His mouse monoclonal antibody (Thermo Fisher) in blocking buffer for 1 h and washed three times with PBS-T. The strip was incubated at RT with 1:3000 anti-mouse goat antibody coupled with horse radish peroxidase (Novex ECL) in a blocking buffer for 1 h and washed three times with PBS-T. Blot was developed by chemiluminescence (Immobilon Classico Western HRP substrate, Millipore).

### Supplementary Information

**Additional file 1: Table S1.** Amino acid sequence similarities of *T. vaginalis *ERMES components with *S. cerevisiae *orthologs.

**Additional file 2: Fig. S1.** Protein sequence alignment of TvMmm1 (A), TvMdm12 (B), and TvMmm2 (C) with model yeast orthologs. **Fig. S2.** Protein sequence alignment of TvMmm1d and TvMmm1e N-terminal domains with parabasalid orthologs. **Fig. S3.** Phylogenetic analysis of Nvj2 and ERMES components Mmm1, Mmm2, and Mdm12. **Fig. S4.** Volcano plot analysis of proteins coIP with ERMES components (baits). **Fig. S5.** Phylogenetic analysis of beta-barrel proteins to investigate the relationship of Porin2 of Parabasalia and Mdm10. **Fig. S6.** Phylogenetic analysis of *T. vaginalis* HSP70 chaperones. **Fig. S7.** Modeling of TvMmm1a homodimer. **Fig. S8.** Hydrophobic and polar interaction of the interface of TvMmm1a-TvMdm12 heterodimer. **Fig. S9.** Superposition of *T. vaginalis* and *Z. rouxii *Mmm1-Mdm12 heterotetramer. **Fig. S10.** Hydrophobic and polar interactions of the interface of TvMdm12-TvMmm2b. **Fig. S11.** Modeling of TvMmm2b-Porin2 interactions. **Fig. S12-15.** Raw immunoblots.

**Additional file 3: Table S2.** ERMES components and lipid metabolizing enzymes in organisms with hydrogenosomes and selected aerobic relatives. Table S2A. Summary of identified genes in organisms with hydrogenosomes, mitosomes and selected aerobic relatives. Table S2B. Summary of identified genes in EukProt TCS database. Table S2C. The list of accession numbers. Table S2D. Cell localization predictions for Psd1 and CLST.

**Additional file 4: Table S3.** Significantly enriched proteins coIP with ERMES baits.

**Additional file 5: Table S4.** ColabFold modeling of ERMES components interactions.

**Additional file 6: Table S5.** AutoDock Vina docking score statistics for TvMdm12-phospholipids docking.

**Additional file 7: Table S6.** List of primers.

## Data Availability

The mass spectrometry proteomics data have been deposited to the ProteomeXchange Consortium via the PRIDE [70] partner repository with the dataset identifier PXD044071.

## Abbreviations

AA: Amino acid
Ab: Antibody
AMOEBAE: Analysis of Molecular Evolution with Batch Entry
BMGE: Block Mapping and Gathering with Entropy
CDP-DAG: Cytidine diphosphate diacylglycerol
CL: Cardiolipin
CLS: Cardiolipin synthase
CLS_T_: Cardiolipin synthase with transferase mechanism
coIP: Co-immunoprecipitation
CTA7: C-tail anchored protein 7
DAG: Diacylglycerol
DSP: Dithiobis(succinimidyl propionate)
ER: Endoplasmic reticulum
ERMES: Endoplasmic reticulum mitochondria encounter structure
FDR: False discovery rate
HA: Hemagglutinin
CHP: Conserved hypothetical protein
IPTG: Isopropyl β-D-1-thiogalactopyranoside
IQR: Inter quartal range
LA: Lysophosphatidic acid
LDV: Light density fraction
LECA: Last eukaryotic common ancestor
LFQ MS: Label-free quantitative mass spectrometry
MCS: Membrane contact sites
ME: Malic enzyme
ML: Maximum likelihood
MOM: Mitochondrial outer membrane
NTS: N-terminal mitochondrial targeting signal
Nvj2: Nucleus–vacuole junction 2 protein
PA: Phosphatidic acid
PBS: Phosphate-buffered saline
PBS-T: Phosphate-buffered saline—Tween 20
PC: Phosphatidylcholine
PCC: Pearson correlation coefficient
PDB: Protein Data Bank
PDI: Protein disulfide-isomerase
PE: Phosphatidylethanolamine
PG: Phosphatidylglycerol
PGPS: Phosphatidylglycerol phosphate synthase
PGPS_T_: Phosphatidylglycerol phosphate synthase with a transferase mechanism
Pis: Phosphatidylinositol synthase
PS: Phosphatidylserine
Psd1: Phosphatidylethanolamine decarboxylase
PtdIns(3,4,5)P3: Phosphatidylinositol 3,4,5-trisphosphate
PtdIns(4)P: Phosphatidylinositol 4-phosphate
PtdIns(4,5)P2: Phosphatidylinositol 4,5-bisphosphate
PtdInsP: Phosphatidylinositol
RT: Room temperature
SAR: Stramenopiles-Alveolata-Rhizaria
SGC: 3-Sulfogalactosylceramide
SIM: Structured illumination microscopy
SMP: Synaptotagmin-like mitochondrial lipid-binding protein
TEAB: Triethylammonium bicarbonate
TG: Triglyceride
TMD: Transmembrane domain
TRIS: Tris(hydroxymethyl)aminomethane
TYM: Trypton-Yeast extract-Maltose
