# Supplementary material for: The divergent ER-mitochondria encounter structures (ERMES) are conserved in parabasalids but lost in several anaerobic lineages with hydrogenosomes
Source: BMC Biol. 2023 Nov 15;21:259. doi: 10.1186/s12915-023-01765-1 (PMC10648710; doi:10.1186/s12915-023-01765-1)

Supplementary Figures

Kučerová et al. 2023, The divergent ER-mitochondria encounter structures (ERMES) are conserved in parabasalids but lost in several anaerobic lineages with hydrogenosomes

Figure S1. Protein sequence alignment of TvMmm1 (A), TvMdm12 (B), and TvMmm2 (C) with model yeast orthologs. Green and red delimited rectangles highlight α-helices and β-strands, respectively. Blue and purple indicate the residuals of the proteins, which interact with their respective interacting partners Mmm1a (homodimer) and Mdm12 (heterodimer), respectively [3]. Asterisks indicate the end of a sequence. Red X numbers represent the number of hidden amino acid residues in the given sequence.

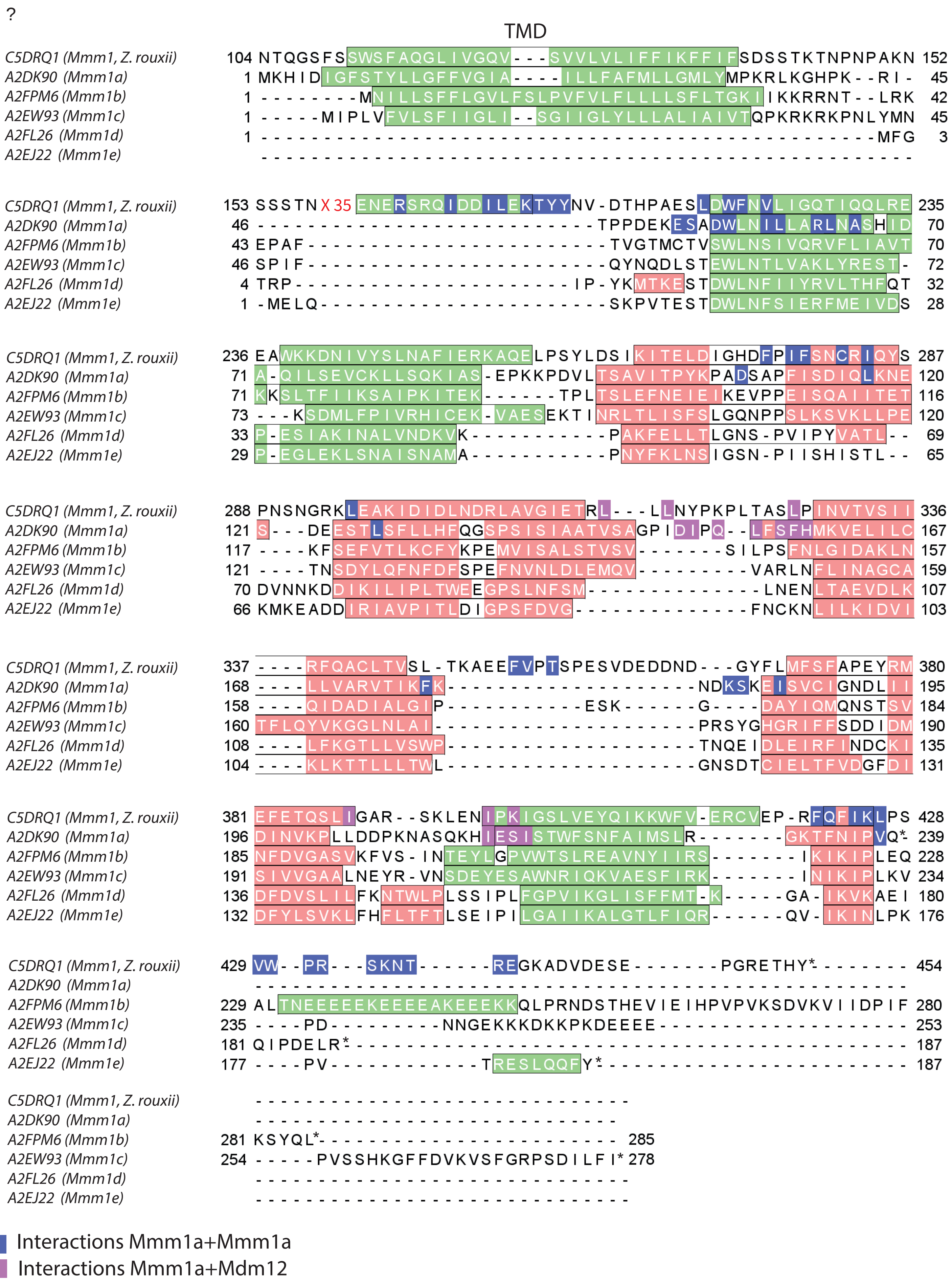

B

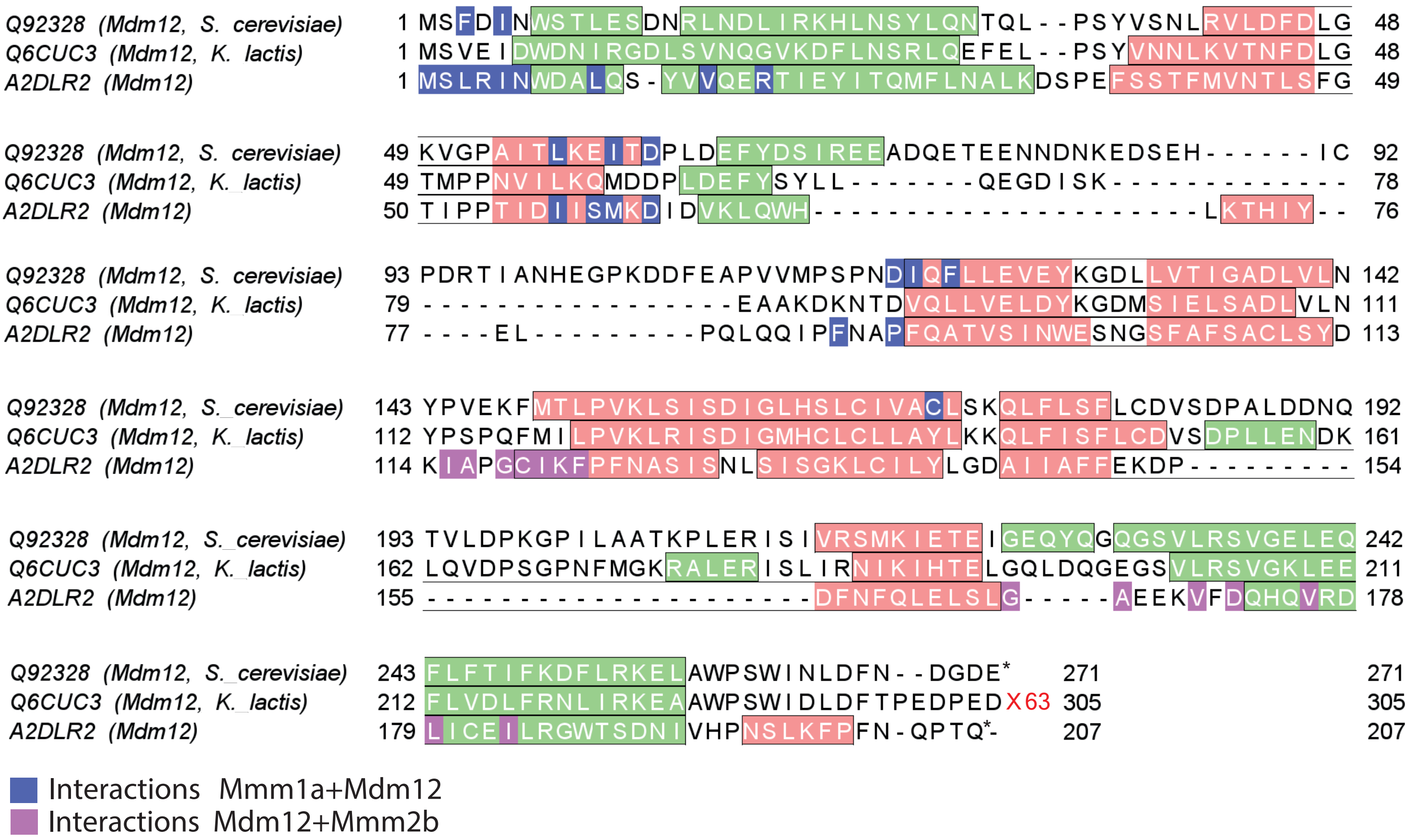
Q92328 (Mdm12, S. cerevisiae) 1 MSFDINWSTLESDNRLNDLIRKHLNSYLQNTQL--PSYVSNLRVLDFDLG 48
Q6CUC3 (Mdm12, K. lactis) 1 MSVEIDWDNIRGDLSVNQGVKDFLNSRLQEFEL--PSYVNNLKVTNFDLG 48
A2DLR2 (Mdm12) 1 MSLRINWDALQS-YVVQERTIEYITQMFLNALKDSPEFSSTFMVNTLSFG 49
Q92328 (Mdm12, S. cerevisiae) 49 KVGPAITLKEITDPLDEFYDSIREEADQETEENNDNKEDSEH------IC 92
Q6CUC3 (Mdm12, K. lactis) 49 TMPPNVILKQMDDPLDEFYSYLL-------QEGDISK-------------- 78
A2DLR2 (Mdm12) 50 TIPPTIDIISMKDIDVKLQWH---------------------LKTHIY-- 76
Q92328 (Mdm12, S. cerevisiae) 93 PDRTIANHEGPKDDFEAPVVMPSPNDIQFLLEVEYKGDLLVTIGADLVLN 142
Q6CUC3 (Mdm12, K. lactis) 79 -----------------EAAKDKNTDVQLLVELDYKGDMSIELSADLVLN 111
A2DLR2 (Mdm12) 77 ----EL----------PQLQQIPFNAPFQATVSINWESNGSFAFSACLSYD 113
Q92328 (Mdm12, S. cerevisiae) 143 YPVEKFMTLPVKLSISDIGLHSLCIVACLSKQLFLSFLCDVSDPALDDNQ 192
Q6CUC3 (Mdm12, K. lactis) 112 YPSPQFMILPVKLRISDIGMHCLCLLAYLKKQLFISFLCDVSDPLLENDK 161
A2DLR2 (Mdm12) 114 KIAPGCIKFPFNASISNLSISGKLCILYLGDAIIAFFEKDP---------- 154
Q92328 (Mdm12, S. cerevisiae) 193 TVLDPKGPILAATKPLERISIVRSMKIETEIGEQYQGQGSVLRSVGELEQ 242
Q6CUC3 (Mdm12, K. lactis) 162 LQVDPSGPNFMGKRALERISLIRNIKIHTELGQLDQGEGSVLRSVGKLEE 211
A2DLR2 (Mdm12) 155 ----------------------DFNFQLELSLG-----AEEKVFDQHQVRD 178
Q92328 (Mdm12, S. cerevisiae) 243 FLFTIFKDFLRKELAWPSWINLDFN--DGDE* 271 271
Q6CUC3 (Mdm12, K. lactis) 212 FLVDLFRNLIRKEAAWPSWIDLDFTPEDPED X63 305 305
A2DLR2 (Mdm12) 179 LICEILRGWTSDNIVHPNSLKFPFN-QPTQ*- 207 207
Interactions Mmm1a+Mdm12
Interactions Mdm12+Mmm2b

C

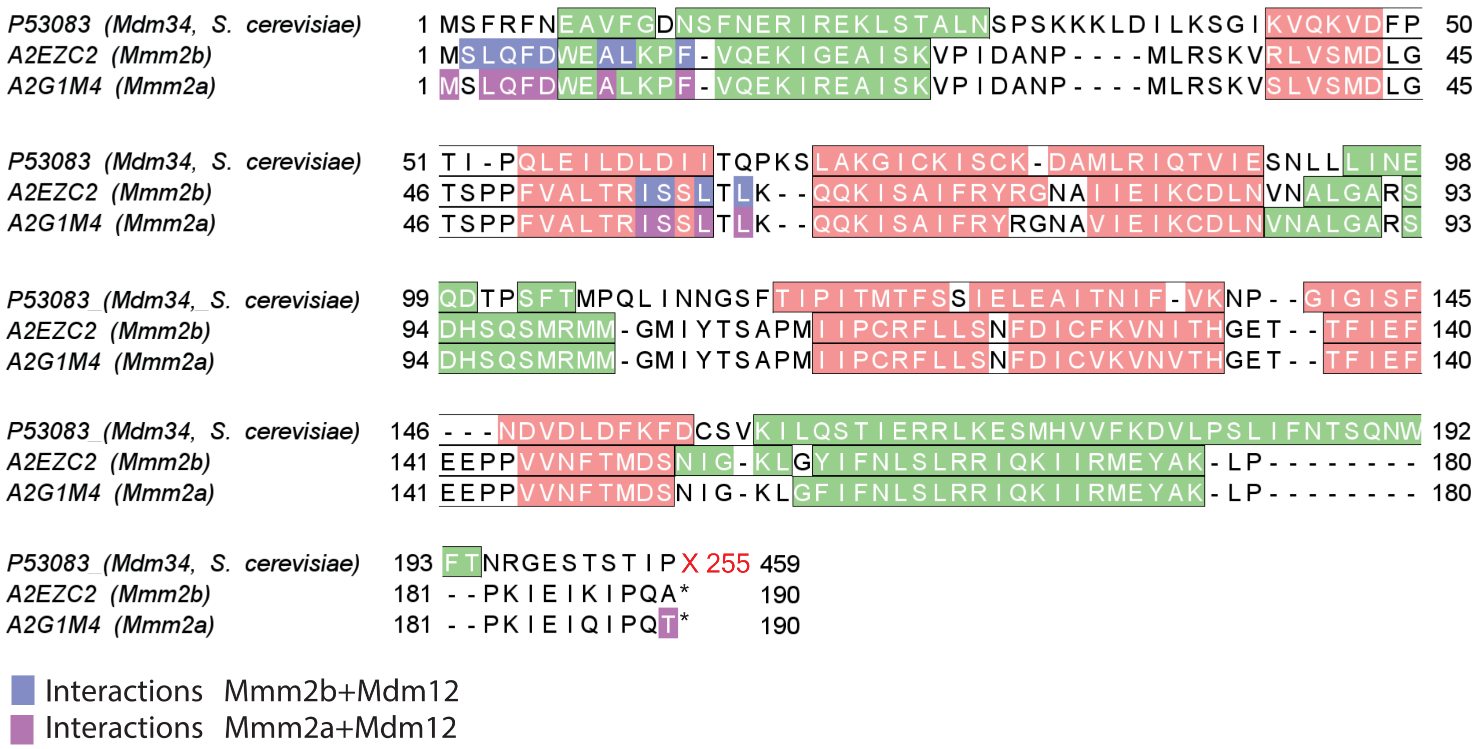
P53083 (Mdm34, S. cerevisiae) 1 MSFRFNEAVFGDNSFNERIREKLSTALNSPSKKKLDILKSGIKVQKVDFP 50
A2EZC2 (Mmm2b) 1 MSLQFDWEALKPF-VQEKIGEAISKVPIDANP----MLRSKVRLVSMDLG 45
A2G1M4 (Mmm2a) 1 MSLQFDWEALKPF-VQEKIREAISKVPIDANP----MLRSKVSLVSMDLG 45
P53083 (Mdm34, S. cerevisiae) 51 TI-PQLEILDLDIITQPKSLAKGICKISCK-DAMLRIQTVIESNLLLINE 98
A2EZC2 (Mmm2b) 46 TSPPFVALTRISSLTLK--QQKISAIFRYRGNAIIEIKCDLNVNALGARS 93
A2G1M4 (Mmm2a) 46 TSPPFVALTRISSLTLK--QQKISAIFRYRGNAVIEIKCDLNVNALGARS 93
P53083 (Mdm34, S. cerevisiae) 99 QDTPSFTMPQLINNGSFTIPITMTFSSIELEAITNIF-VKNP--GIGISF 145
A2EZC2 (Mmm2b) 94 DHSQSMRMM-GMIYTSAPMIIPCRFLLSNFDICFKVNITHGET--TFIEF 140
A2G1M4 (Mmm2a) 94 DHSQSMRMM-GMIYTSAPMIIPCRFLLSNFDICVKVNVTHGET--TFIEF 140
P53083 (Mdm34, S. cerevisiae) 146 ---NDVDLDFKFDCSVKILQSTIERRLKESMHVVFKDVLPSLIFNTSQNW 192
A2EZC2 (Mmm2b) 141 EEPPVVNFTMDSNIG-KLGYIFNLSLRRIQKIIRMEYAK-LP-------- 180
A2G1M4 (Mmm2a) 141 EEPPVVNFTMDSNIG-KLGFIFNLSLRRIQKIIRMEYAK-LP-------- 180
P53083 (Mdm34, S. cerevisiae) 193 FTNRGESTSTIPX 255 459
A2EZC2 (Mmm2b) 181 --PKIEIKIPQA* 190
A2G1M4 (Mmm2a) 181 --PKIEIQIPQT* 190
Interactions Mmm2b+Mdm12
Interactions Mmm2a+Mdm12

Figure S2. Protein sequence alignment of TvMmm1d and TvMmm1e N-terminal domains with parabasalid orthologs. Green rectangles highlight the transmembrane domain (TMD) predicted with TMHMM 2.0 [91]. Stars indicate incomplete sequences.

```
                                                 TMD
Mmm1d_T.vaginalis_XP_001307327.1    ----------------------------------MFGTRP---IPYKM -11
Mmm1d_T.foetus_OHS95838.1           --MNSFFIGFLFSFISLLVILVILRLTVPYASKLFGNKP---IPYKS -42
Mmm1d_T.gallinae_k48_S337288.p1     ------*LTGFVFTFSILLILLIVLRLTIPFASRIFGTRP---IPYKF -38
Mmm1d_T.tenax_k64_S111599.p1       *KTMGPFLAGFLFTFFLLLIALIFLRLTIPFASKLFGTRP---IPYKF -44
Mmm1d_P.hominis_k48_S211708.p1      ---*NPFFLGFLISFSLVFIVIIALRLTVPYASRLFGTTP---IPYKS -41
Mmm1d_T.gallinarum_TEGa034544       -----*FFLGFLISFSLVFIVIIALRLTVPYASRLFGTTP---IPYKS -39
Mmm1d_H.meleagridis_KAH0796090.1    --MGAFYNGFVFTLVAILIFILFML--FYYFDKLEKQNQLLVDLSKA -43
Mmm1d_D.fragilis_k48_S1444524.p1   *RKMGSIMKGYLFGQILFLIAVVYL-----YYRRICQHPR---RLKKA -52
Mmm1e_T.vaginalis_XP_001319548.1    --------------------------------------ME---LQSKP -7
Mmm1e_T.tenax_k48_S86699.p1         ----*LFYACF-KYIFIFLLILLVLRGAIPFIVKIIPPLE---LTSKP -39
Mmm1e_T.gallinae_k32_S528171.p1     ----*LFYVCF-KCIFIFLLILLVLRGVIPFIAKVIPPME---LTTKP -39
Mmm1e_P.hominis_k48_R708168.p1      ----*GFSTYFWKTLYLLSILIIITRCILAILKRYFRPKQ---SPKIP -40
Mmm1e_T.gallinarum_TEGa021766       --------------*YLLSILIIITRCILAILKRYFRPKQ---SPKIP -30
```

Figure S3. Phylogenetic analysis of Nvj2 and ERMES components Mmm1, Mmm2, and Mdm12. The maximum likelihood (ML) tree was constructed using IQ-TREE (Best fit; LG+F+G4 model) with 61 sequences and 331 sites. Bootstrap support values and aBayes posterior probability were calculated using 1000 replicates each. The support values are represented in the order of aBayes (Posterior probability value)/ML ultra-fast bootstrapping/ML non-parametric bootstrapping.

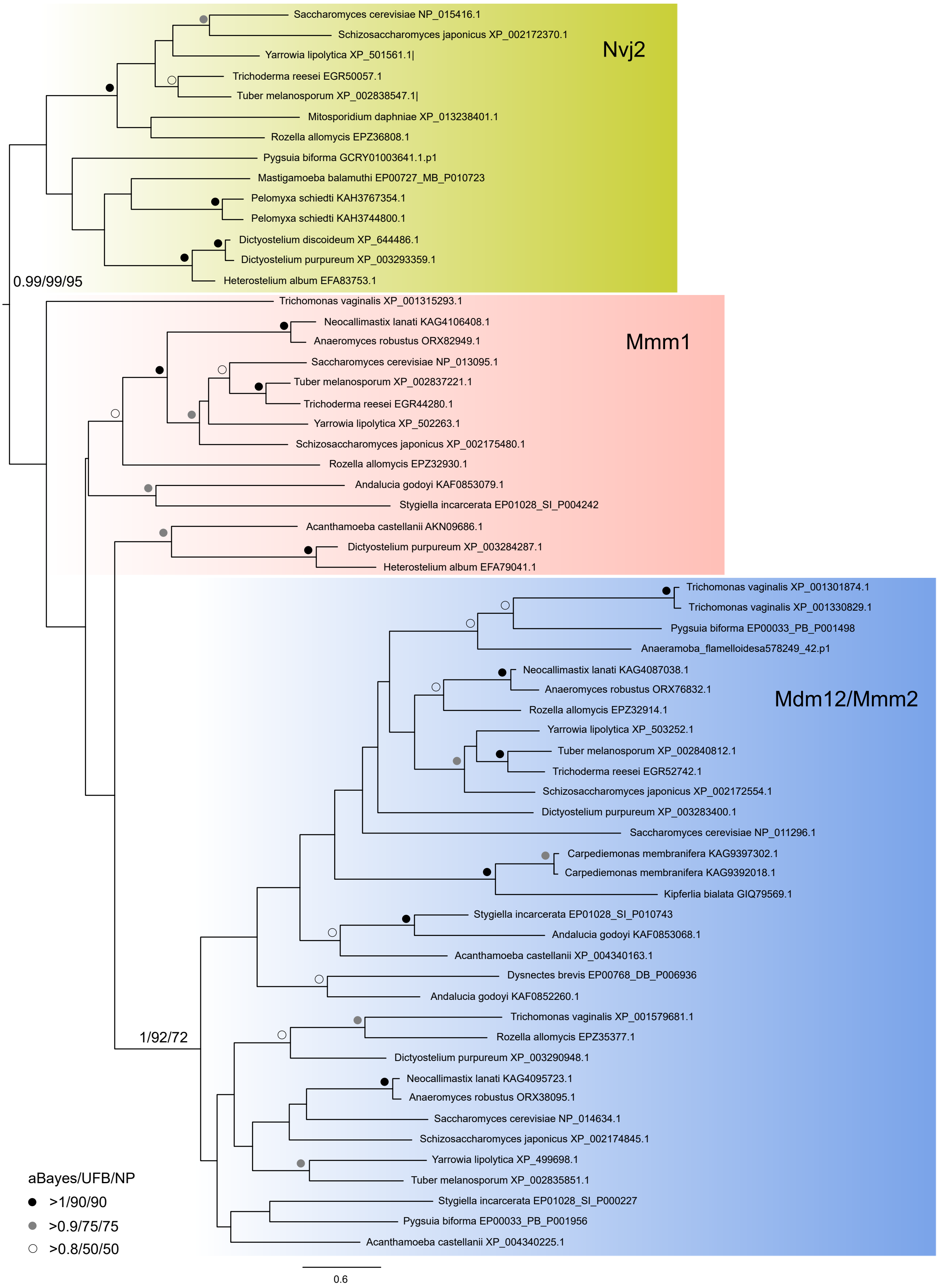

Figure S4. Volcano plot analysis of proteins coIP with ERMES components (baits). The vertical axis corresponds to the mean value of -log10 p-value and the horizontal axis displays the corresponding t-test difference. Red dots represent baits, each was used in three independent coIP experiments. Black dots represent proteins that were used for interactome construction (Fig. 6). The cut-off curve, assigning significant potential interactors, is based on the false discovery rate (FDR=0.05) and the artificial factor s0 (s=1).

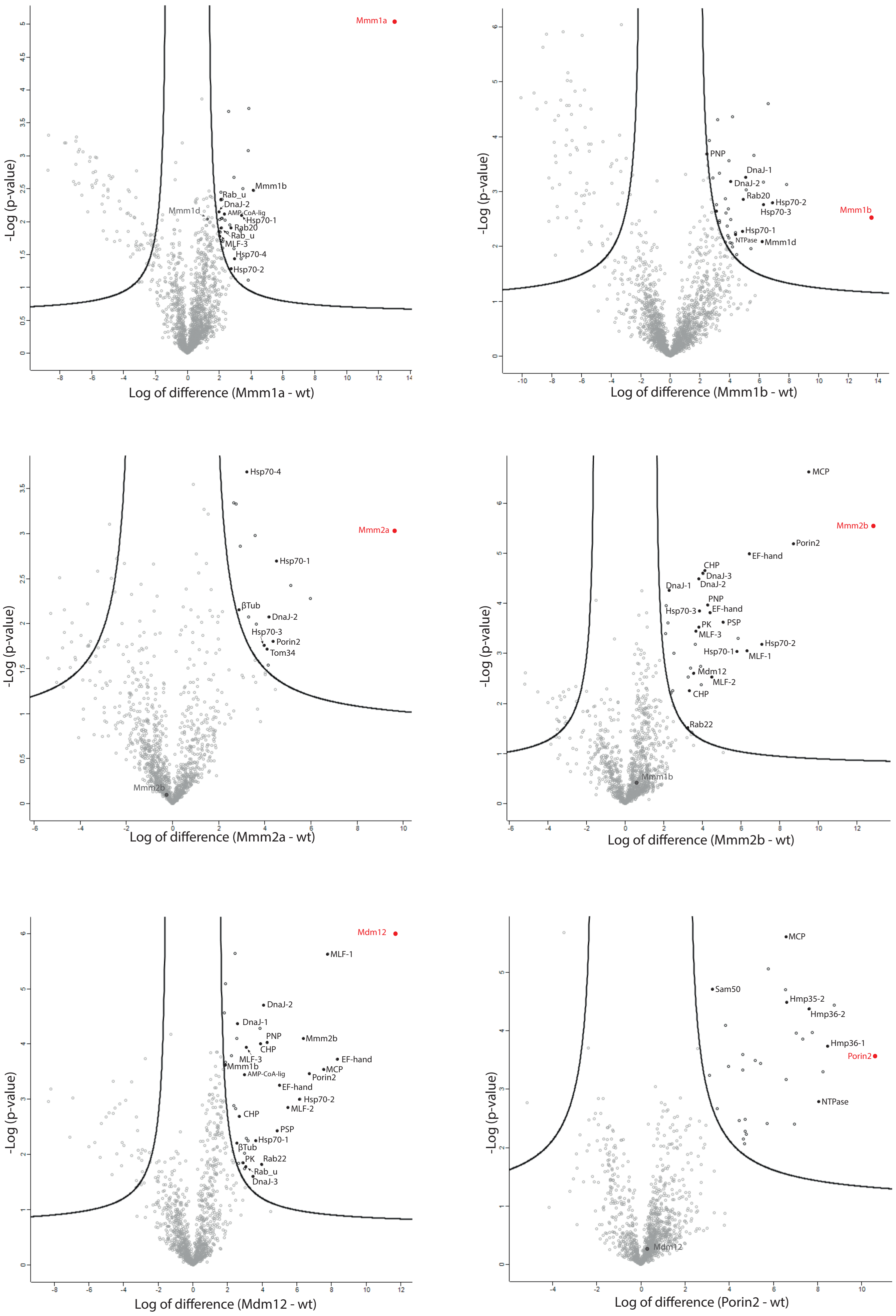

Mmm1a
Mmm1b
Rab_u
DnaJ-2
Mmm1d
AMP-CoA-lig
Hsp70-1
Rab20
Rab_u
MLF-3
Hsp70-4
Hsp70-2
-Log (p-value)
Log of difference (Mmm1a - wt)
PNP
DnaJ-1
DnaJ-2
Rab20
Hsp70-2
Hsp70-3
Mmm1b
Hsp70-1
NTPase
Mmm1d
-Log (p-value)
Log of difference (Mmm1b - wt)
Hsp70-4
Mmm2a
Hsp70-1
βTub
DnaJ-2
Hsp70-3
Porin2
Tom34
Mmm2b
-Log (p-value)
Log of difference (Mmm2a - wt)
MCP
Mmm2b
Porin2
EF-hand
CHP
DnaJ-3
DnaJ-1
DnaJ-2
PNP
Hsp70-3
EF-hand
PK
PSP
MLF-3
Hsp70-2
Hsp70-1
MLF-1
Mdm12
MLF-2
CHP
Rab22
Mmm1b
-Log (p-value)
Log of difference (Mmm2b - wt)
Mdm12
MLF-1
DnaJ-2
DnaJ-1
PNP
Mmm2b
CHP
MLF-3
EF-hand
Mmm1b
MCP
AMP-CoA-lig
Porin2
EF-hand
Hsp70-2
MLF-2
CHP
PSP
Hsp70-1
βTub
PK
Rab22
Rab_u
DnaJ-3
-Log (p-value)
Log of difference (Mdm12 - wt)
MCP
Sam50
Hmp35-2
Hmp36-2
Hmp36-1
Porin2
NTPase
Mdm12
-Log (p-value)
Log of difference (Porin2 - wt)

Figure S5. Phylogenetic analysis of beta-barrel proteins to investigate the relationship of Porin2 of Parabasalia and Mdm10. The maximum likelihood tree was constructed using IQ-TREE (Best fit; Q.pfam+F+I+G4 model) with 67 sequences and 237 sites. Porins in blue represent previously analyzed sequences [13]. The Tom40 clade in pink was used as an outgroup. Ultrafast bootstrap support (UFB) values, and Standard non-parametric (NP) bootstrap support values were calculated using 1000 replicates, and 100 replicates, respectively. aBayes Posterior probability values were calculated using 100 replicates. The support values are represented in the order of aBayes/UFB/NP. Support values below 0.5 (aBayes) and 50 (UFB/NP) are omitted or represented by a dash (-), while nodes with a support value of 100 for both Ultrafast and NP bootstrapping and 1 for posterior probability are represented with black solid triangles.

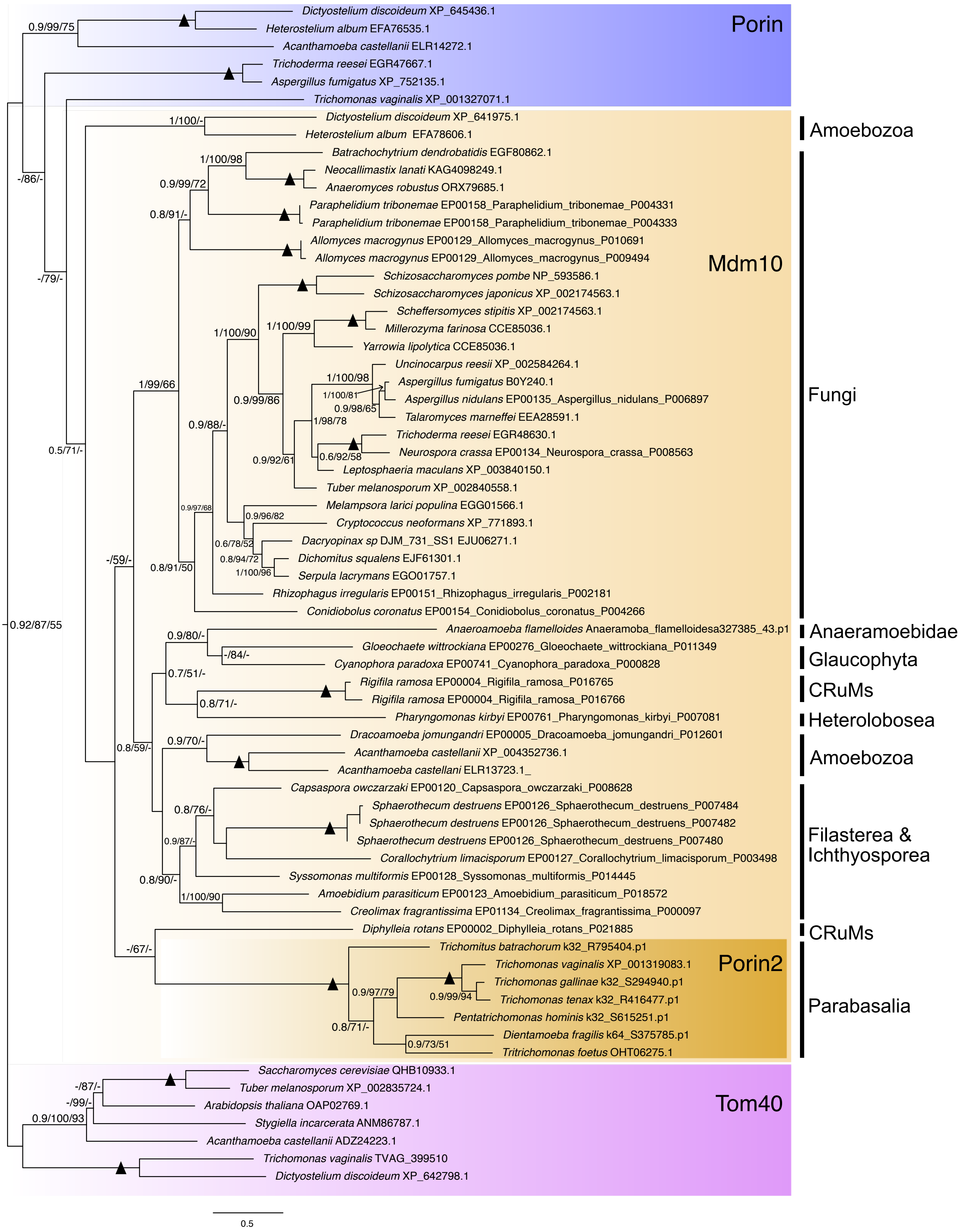

Figure S6. Phylogenetic analysis of *T. vaginalis* HSP70 chaperones. The maximum likelihood tree was constructed using IQ-TREE (LG+F+I+G4 model) with 41 protein sequences and 367 sites. Bootstrap support value was calculated using 1000 bootstraps. Red, blue, and orange dots indicate proteins identified in proteomes of *T. vaginalis* lysosomes, hydrogenosomes, and extracellular vesicles, respectively [92][34][93][94][41]. Green dots indicate HSP70 that coimmunoprecipitated with ERMES components.

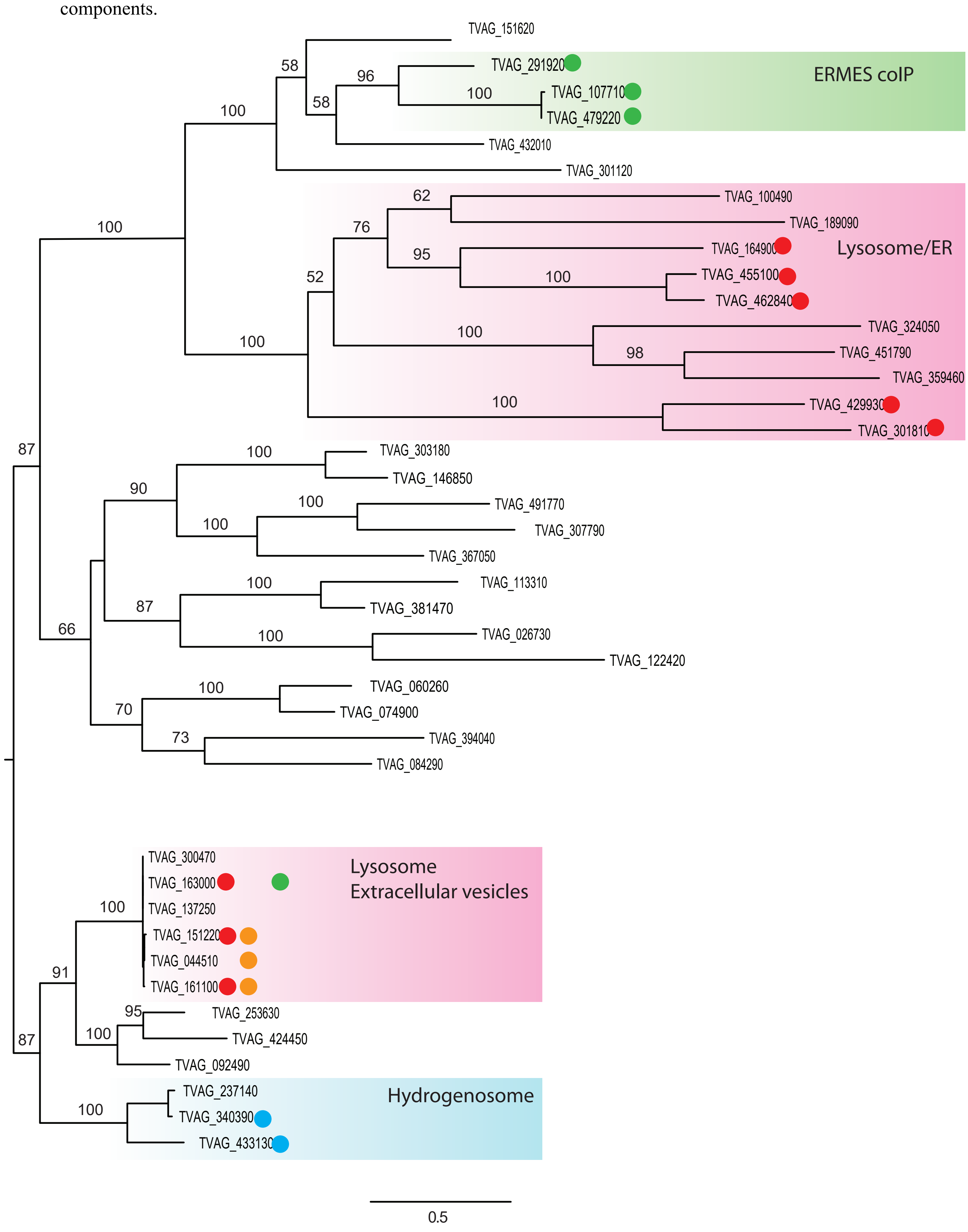

Figure S7. Modeling of TvMmm1a homodimer.

A. Polar contacts were predicted using the Pymol "show_contacs.py" script: good polar interactions (proper atoms, distance, and angle) are shown in yellow and not-ideal contacts are marked in purple.

B. Hydrophobic interactions (defined as hydrophobic amino acids within 4 Å of each other) are marked in blue. Specifically, the prediction indicated hydrophobic interactions of L117, L127, F177, I185, V238, I267, L60, L64 and A66 of green TvMmm1a monomer with I59, L60, L64, A66, L84, L127, F117, I185 and V238 of blue TvMmm1a monomer. The alpha helices and beta sheets forming the SMP domain are numbered from the N terminus.

A

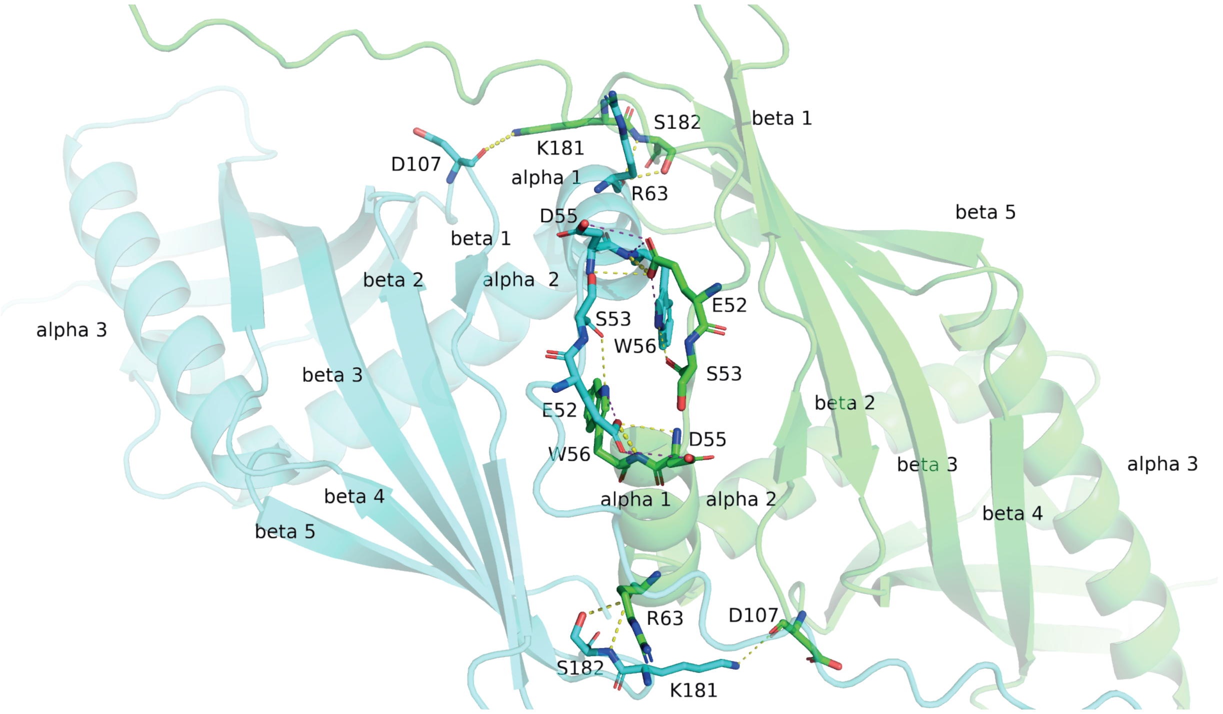

B

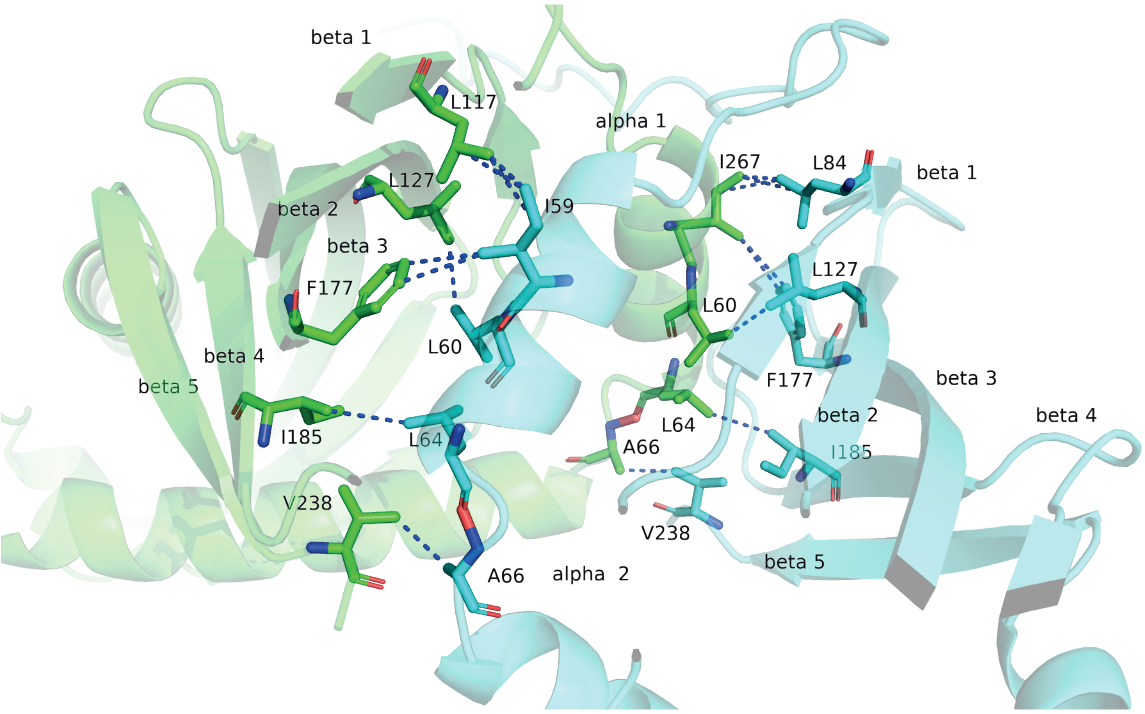

Figure S8. Hydrophobic and polar interaction of the interface of TvMmm1a-TvMdm12 heterodimer.

A. Hydrophobic interactions (defined as hydrophobic amino acids within 4 Å of each other) are marked in blue. Specifically, the prediction indicated hydrophobic interactions of L3, I5, L10, and V15 of TvMdm12 with I152, L155, F156 and F158 of TvMmm1a, respectively. The alpha helices and beta sheets forming the SMP domain are numbered from the N terminus.
B. Polar contacts were predicted by the “show_contacs.py” Pymol script: good polar interactions (proper atoms, distance, and angle) are shown in yellow and not ideal contacts are marked in purple.

A

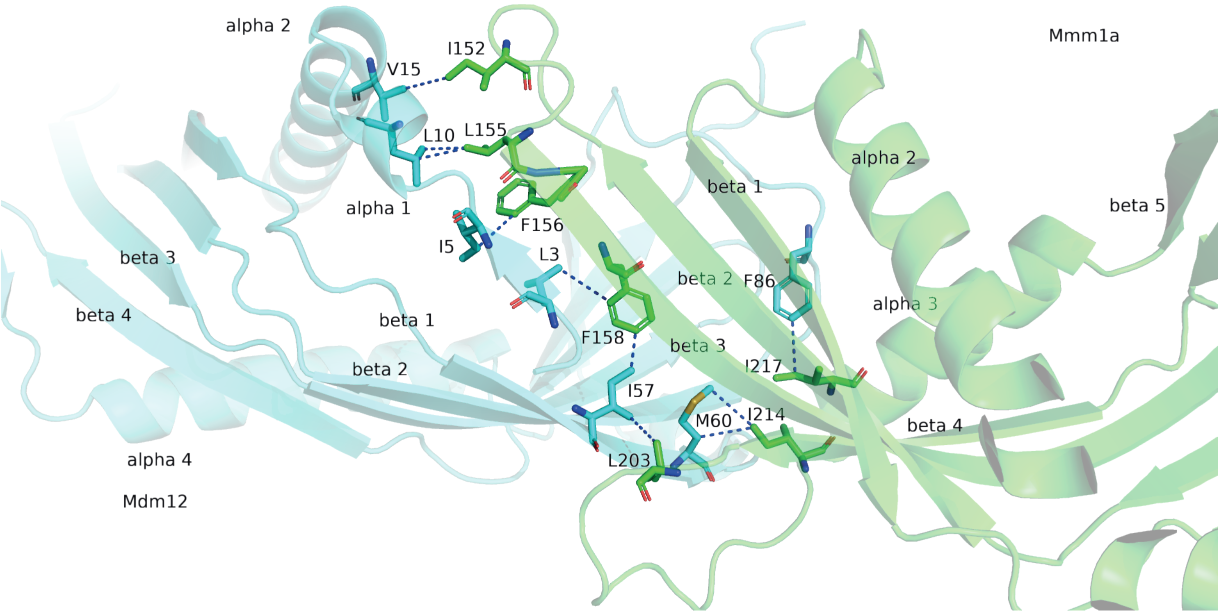

B

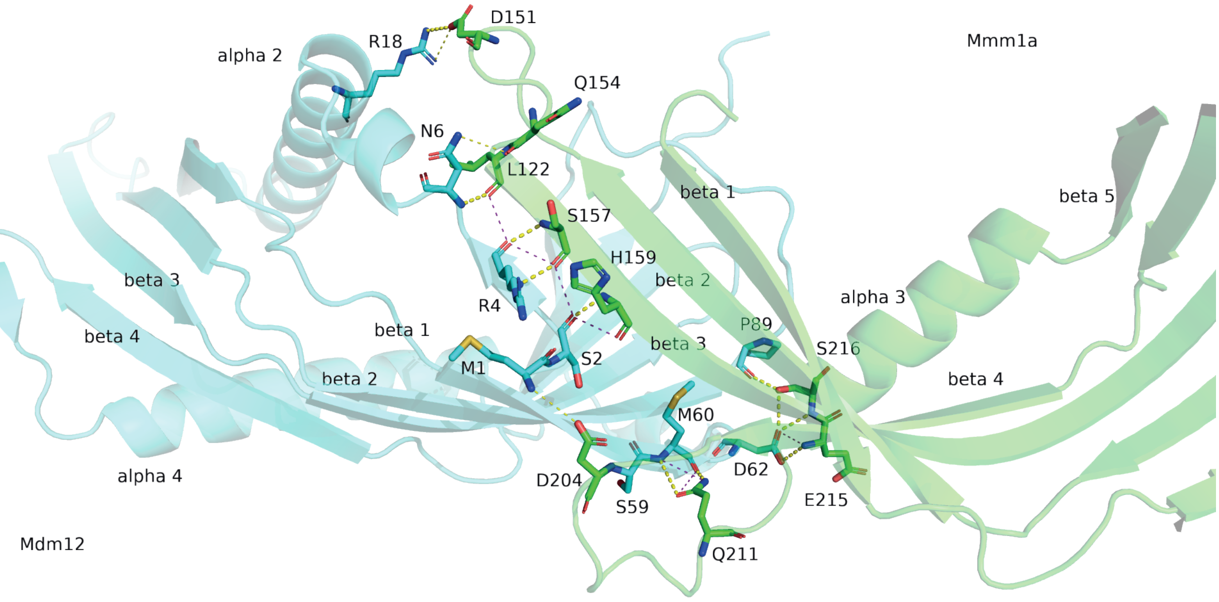

Figure S9. Superposition of *T. vaginalis* and *Z. rouxii* Mmm1-Mdm12 heterotetramer. Crystal Structure of Mdm12-Mmm1 complex (PDB ID 5YK7) and ColabFold predicted *T. vaginalis* heterotetramer (Table S4) were used for the analysis. The red lines indicate axes of each displaced dimer of both tetramers. These axes form the displacement angle of 56.4°.

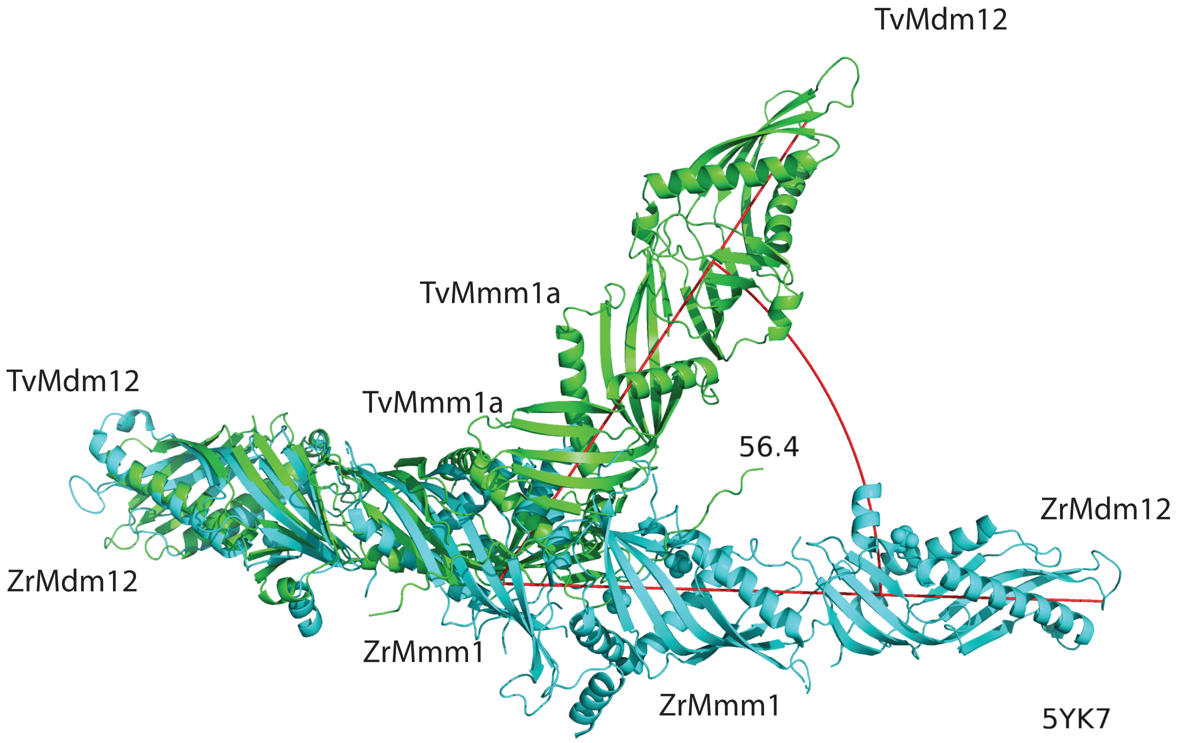

Figure S10. Hydrophobic and polar interactions of the interface of TvMdm12-TvMmm2b.

A. Hydrophobic interactions (defined as hydrophobic amino acids within 4 Å of each other) are marked in blue. Specifically, the prediction indicated hydrophobic interactions of F13, A9, L10, F5, L3, I56, L59, L61 of TvMmm2b with I115, A116, I120, F122, V170, V 176, L179 and I183 of TvMdm12. The alpha helices and beta sheets forming the SMP domain are numbered from the N terminus.

B. Polar contacts were predicted by the “show_contacs.py” Pymol script: good polar interactions (proper atoms, distance and angle) are shown in yellow and not ideal contacts are marked in purple.

A

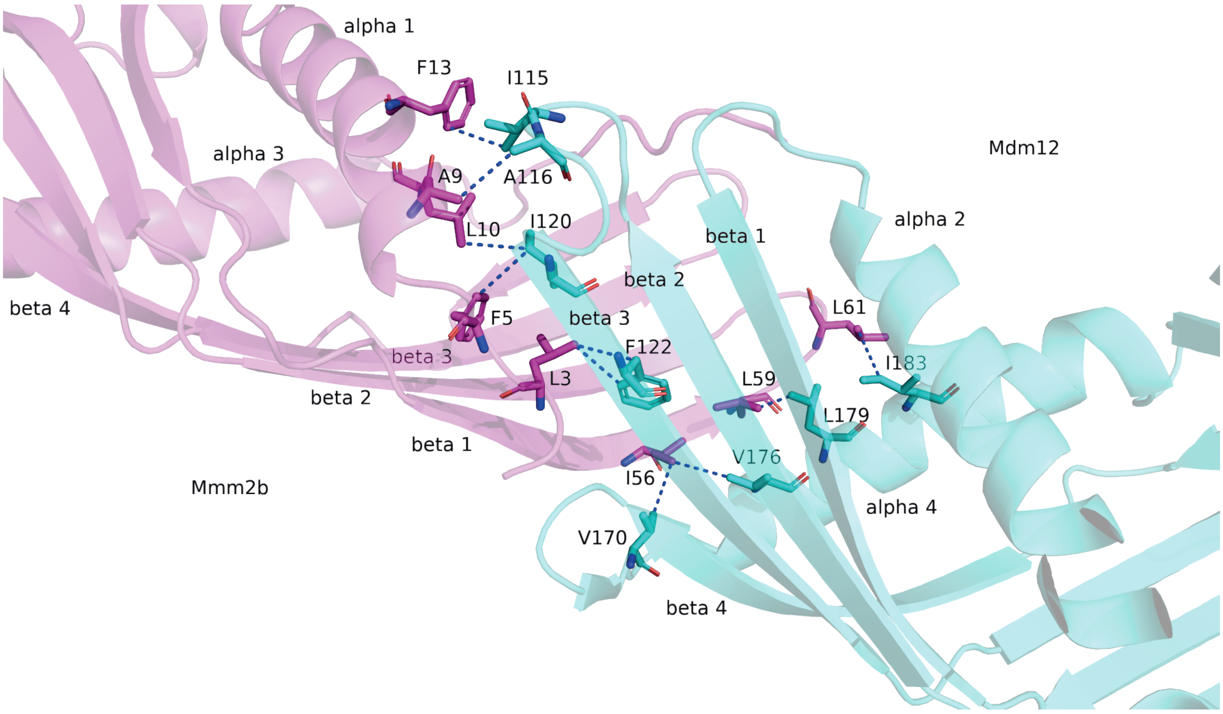

B

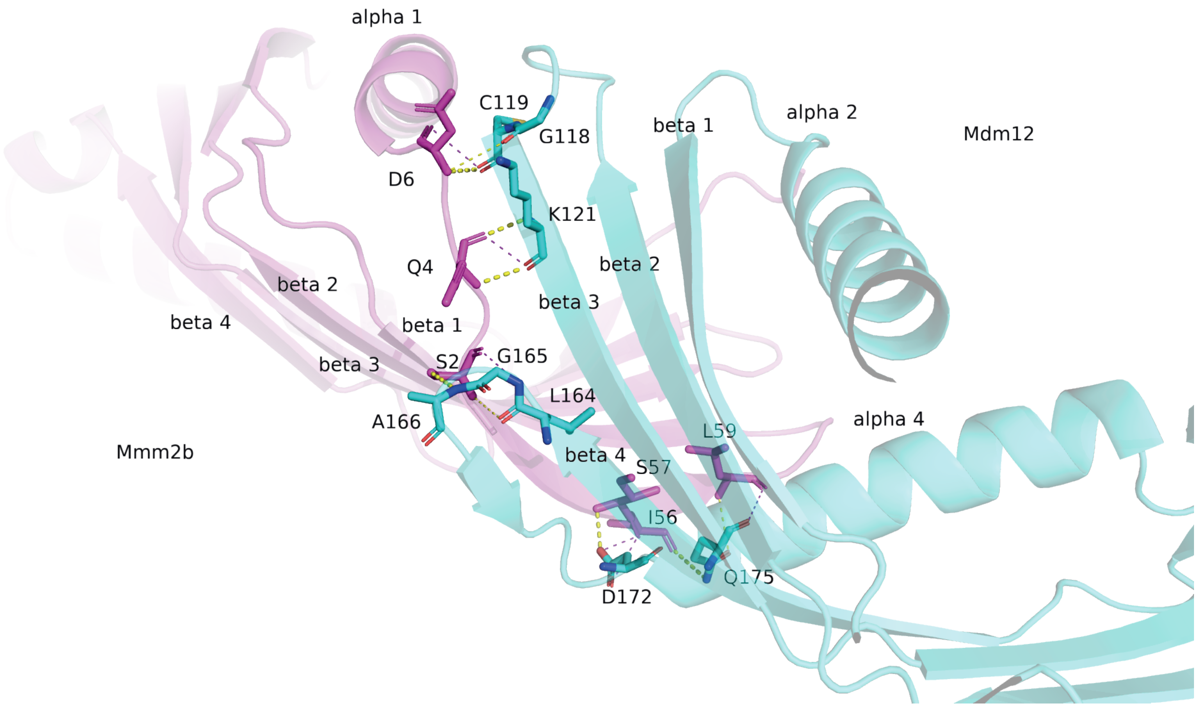

Figure S11. Modeling of TvMmm2b-Porin2 interactions. TvMmm2b α2-helix which is buried in Porin2 is labeled in red.

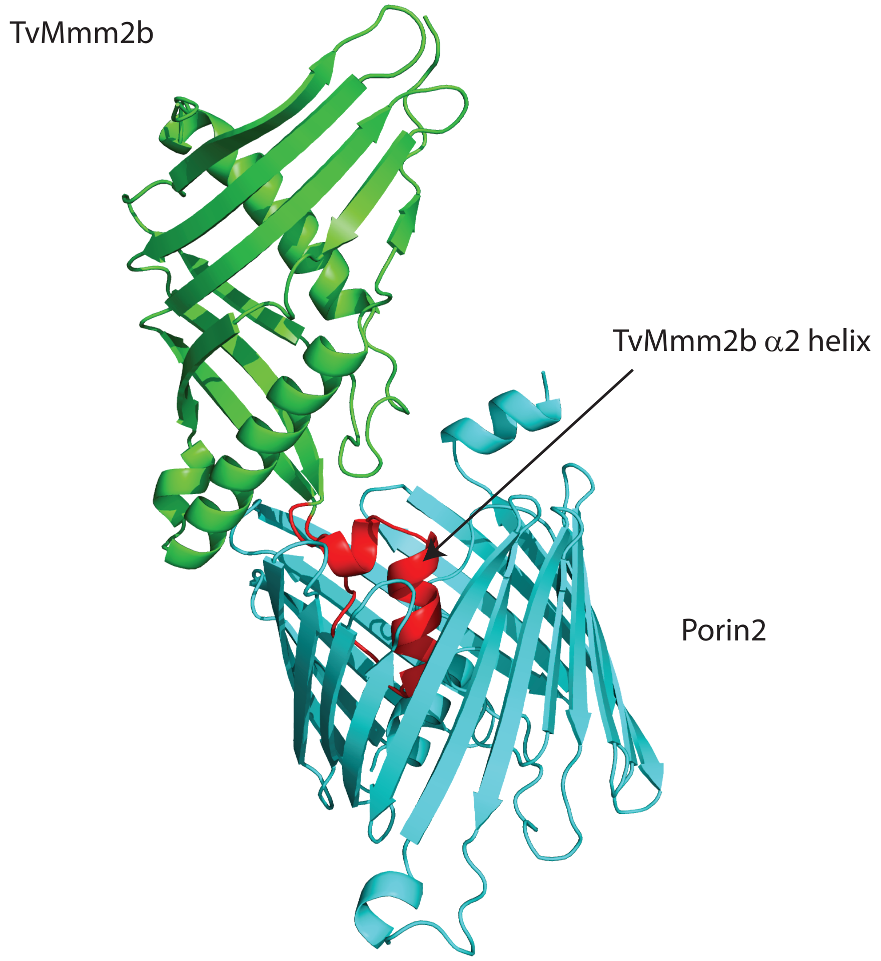

Figure S12. Full immunoblots presented in Figures 5A and 5B.

T. vaginalis cells expressing TvMmm1a or TvMdm12 were sonicated, and subcellular fractions were sepa-rated using differential and Percoll gradient centrifugation. L, cell lysate; C, cytosolic fraction; LDV, low-density vesicles; H, hydrogenosomal fraction. TvMmm1a and TvMdm12 were detected using mouse α-HA antibodies. Cyt. ME, cytosolic malic enzyme (cytosolic marker), OsmC (hydrogenosomal marker), PDI (ER marker) were visualized by mouse α-cyt. ME, rat α-OsmC, and rat α-PDI polyclonal antibodies, respectively. Orange rectangles indicate cropped areas.

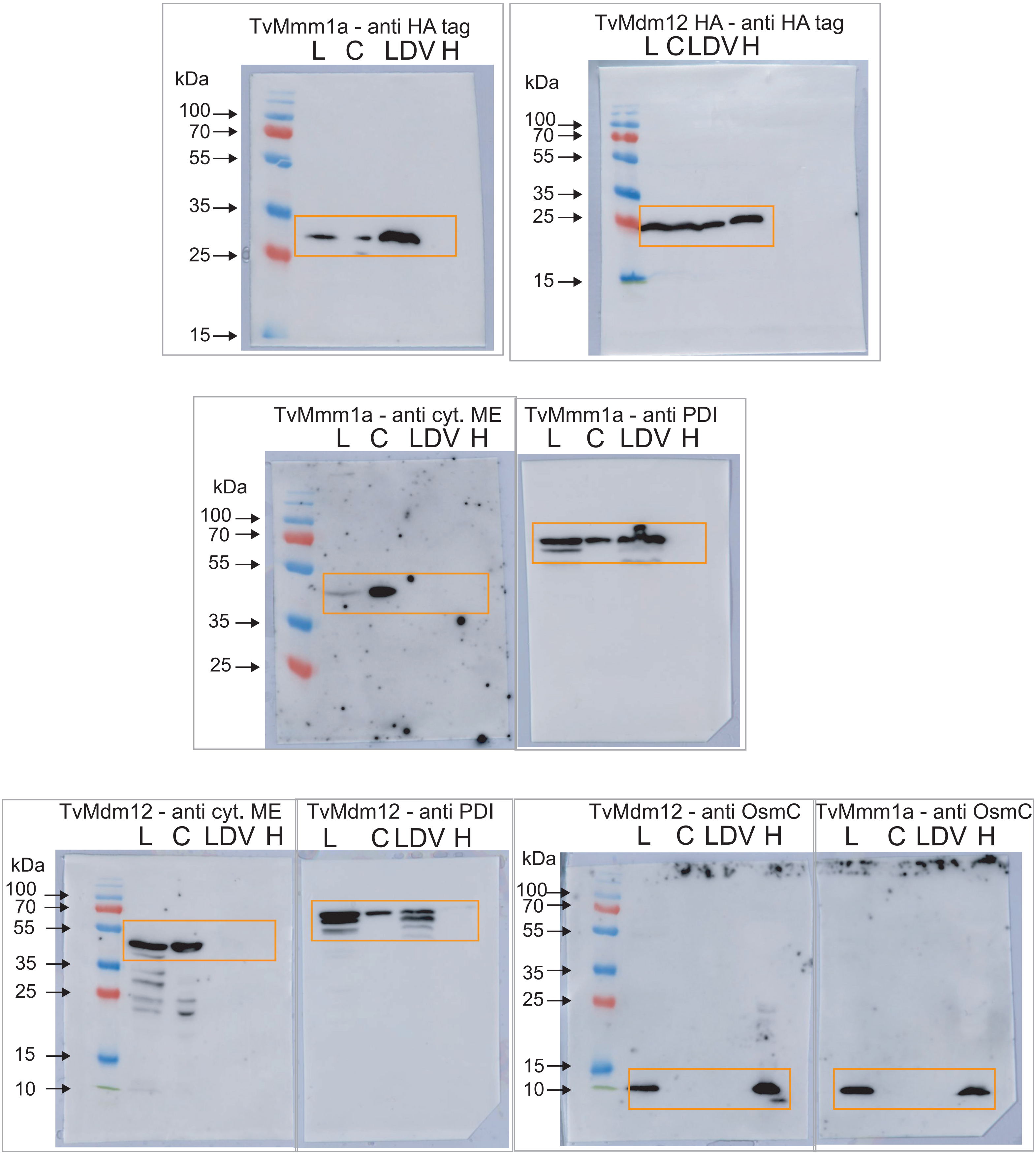

Figure S13. Full immunoblots presented in Figure 5C.

T. vaginalis cells expressing TvMmm2a or TvMmm2b were sonicated, and subcellular fractions were separated using differential and Percoll gradient centrifugation. L, cell lysate; C, cytosolic fraction; LDV, low-density vesicles; H, hydrogenosomal fraction. TvMmm2a and TvMmm2b were detected using mouse α-HA antibodies. Cyt. ME, cytosolic malic enzyme (cytosolic marker), OsmC (hydrogenosomal marker), PDI (ER marker) were visualized by mouse α-cyt. ME, rat α-OsmC, and rat α-PDI polyclonal antibodies, respectively. Orange rectangles indicate cropped areas.

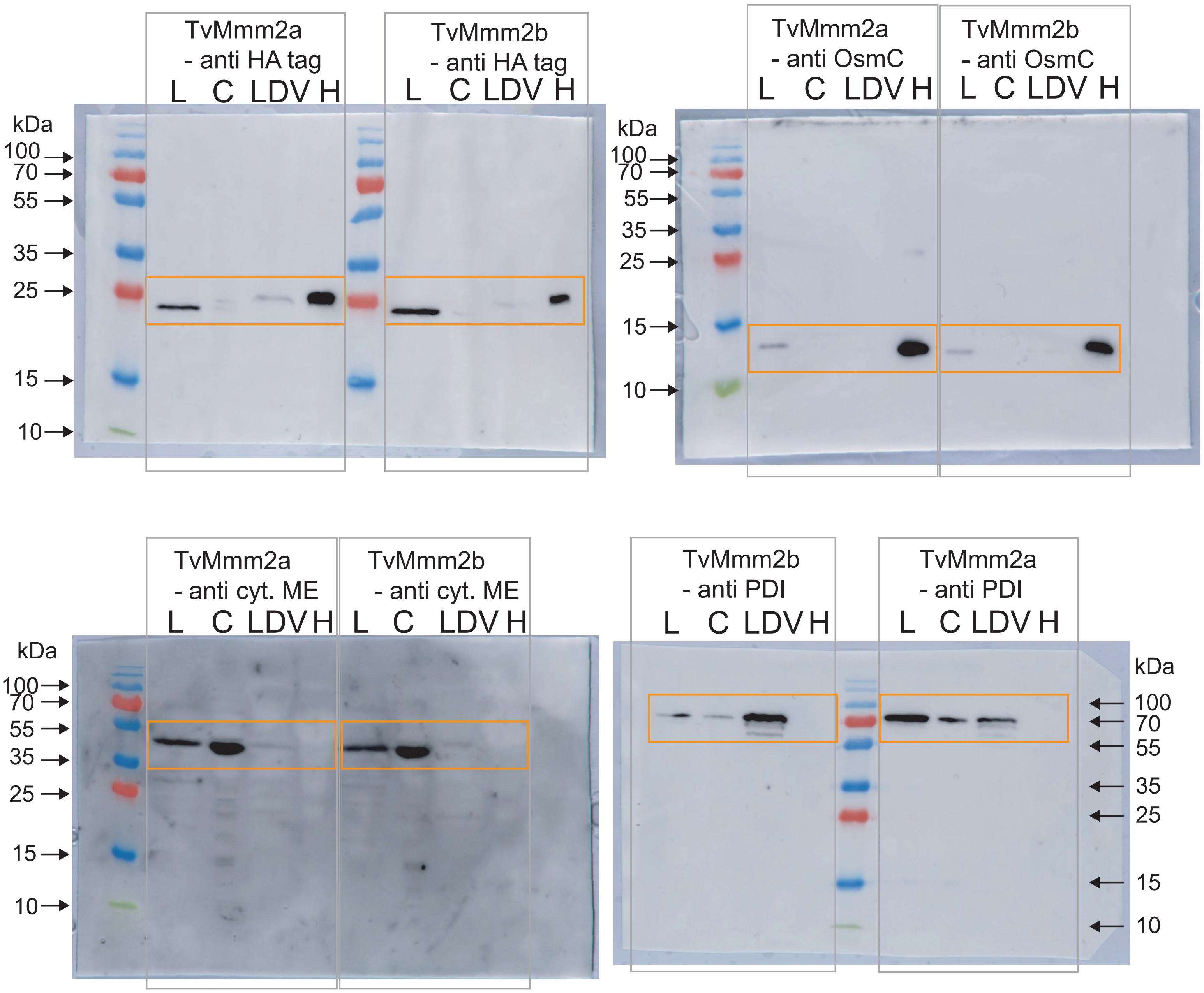

Figure S14. Full immunoblots presented in Figure 5D.

TvMmm2a and TvMmm2b hydrogenosomal fraction (H) was treated with proteinase K (HpK) and proteinase K with Triton X-100 (HpKTX). TvMmm2a and TvMmm2b were detected by mouse α-HA antibody, OsmC (hydrogenosomal marker) was detected by rat polyclonal α- OsmC antibody, and C-tail anchored protein 7 (CTA7, outer hydrogenosomal membrane marker protein) was detected by rat polyclonal α-CTA7 antibody. Orange rectangles indicate cropped areas.

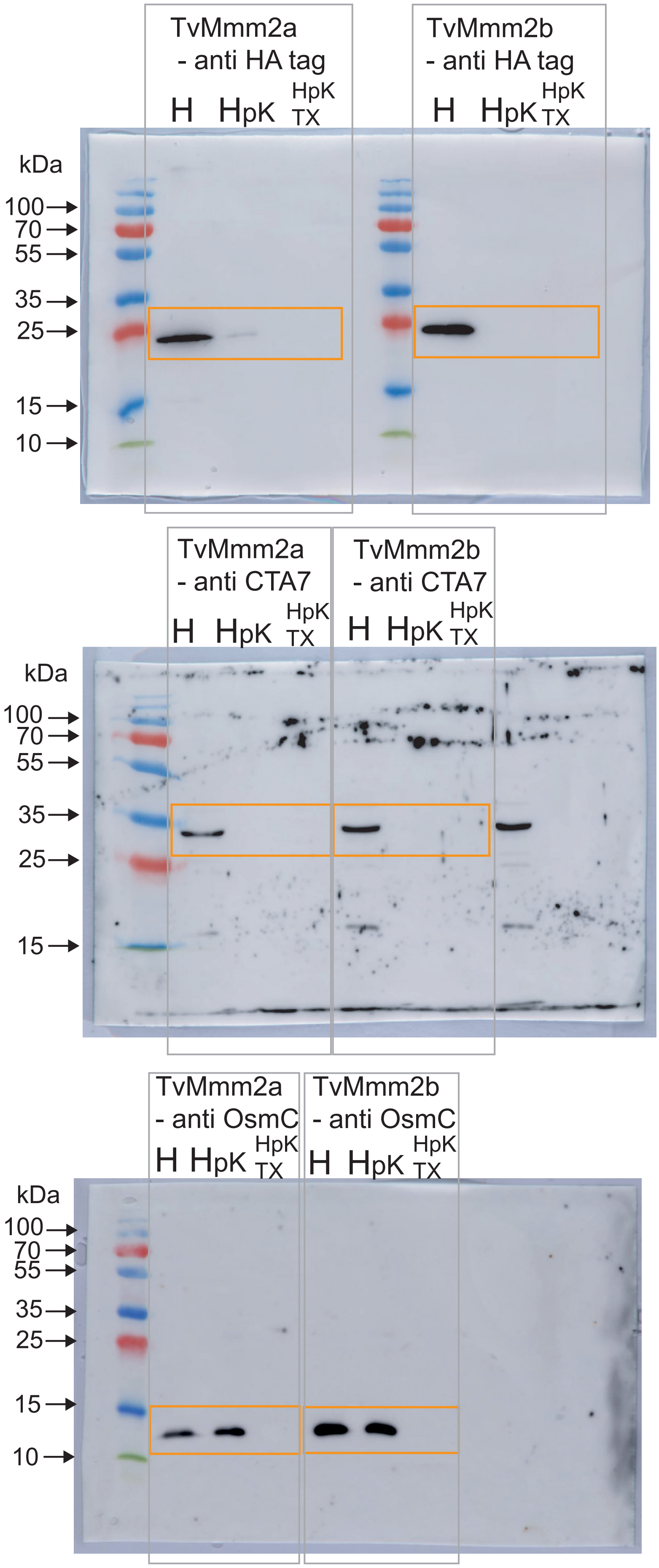

Figure S15. Full immunoblots presented in Figure 7B.
T. vaginalis cells expressing Porin2 were sonicated, and subcellular fractions were separated using differential and Percoll gradient centrifugation. L, cell lysate; C, cytosolic fraction; LDV, low-density vesi-cles; H, hydrogenosomal fraction. Hydrogenosomal fraction (H) was treated with proteinase K (HpK) and proteinase K with Triton X-100 (HpKTX). Porin2 was detected using mouse α-HA antibodies. OsmC (hydrogenosomal marker), C-tail anchored protein 7 (CTA7, outer hydrogenosomal membrane marker protein), PDI (ER marker), and cytosolic malic enzyme (cyt. ME, cytosolic marker) were visualized by rat α-OsmC, rat α-PDI polyclonal, rat α-CTA7, and mouse α-cyt. ME antibodies, respectively. Orange rectangles indicate cropped areas.

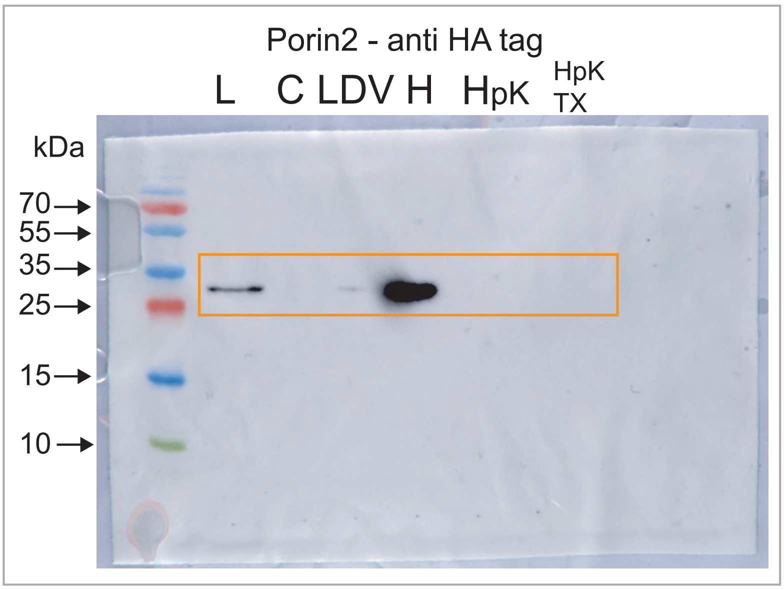

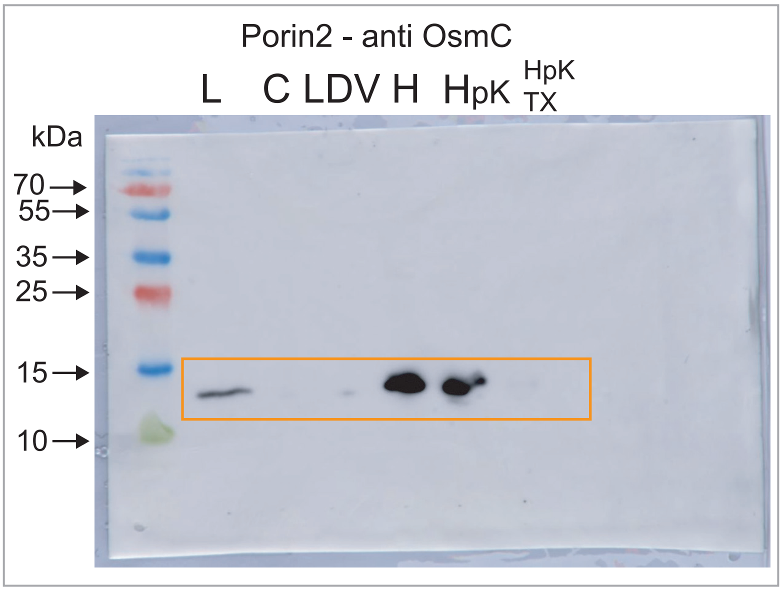

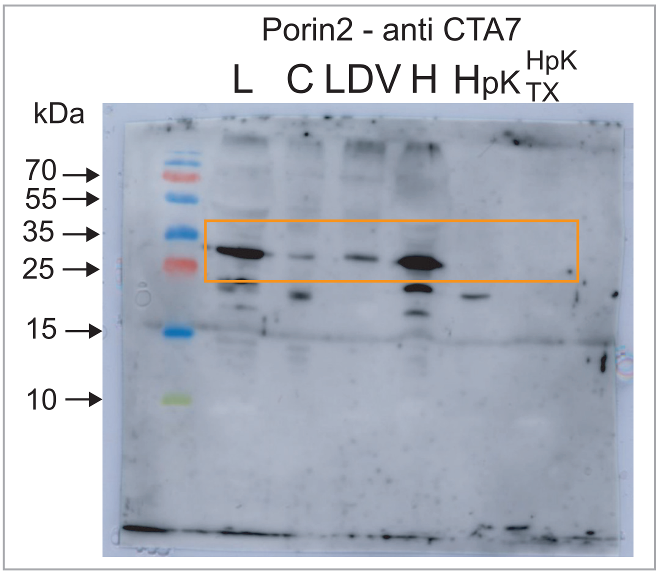

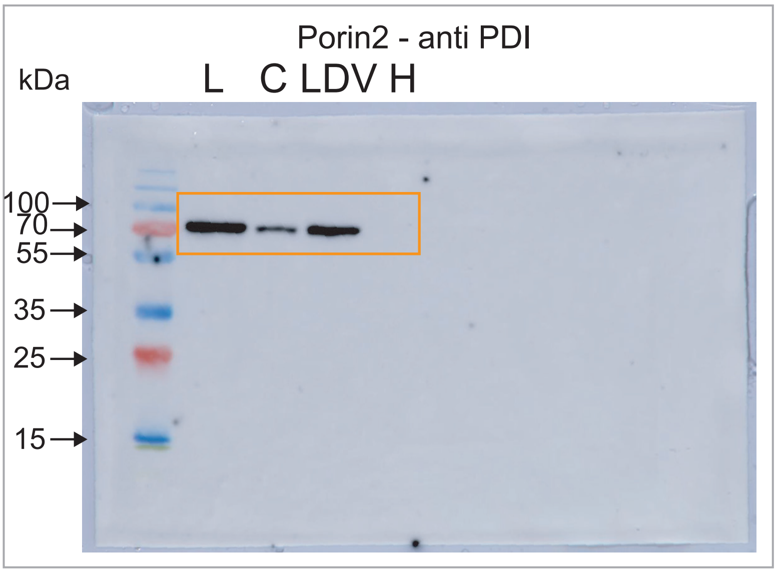

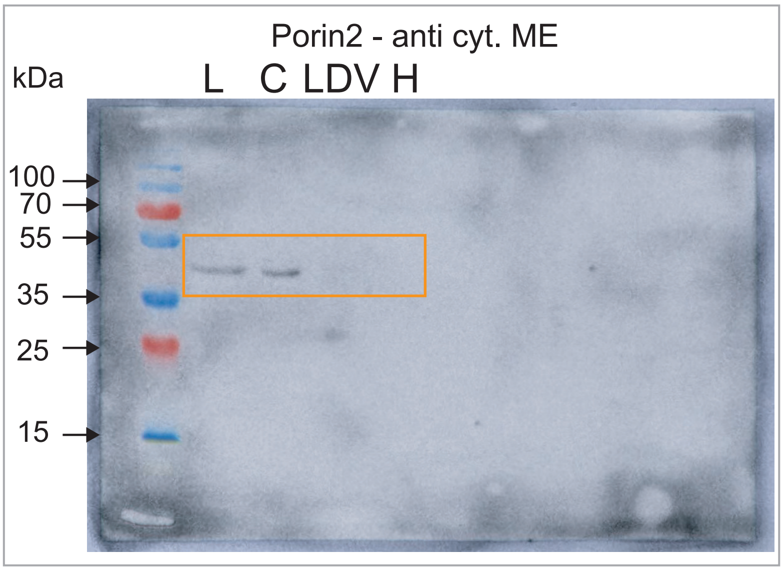

Supplement: Supplementary file 2 — Additional file 2: Fig. S1. Protein sequence alignment of TvMmm1 (A), TvMdm12 (B), and TvMmm2 (C) with model yeast orthologs. Fig. S2. Protein sequence alignment of TvMmm1d and TvMmm1e N-terminal domains with parabasalid orthologs. Fig. S3. Phylogenetic analysis of Nvj2 and ERMES components Mmm1, Mmm2, and Mdm12. Fig. S4. Volcano plot analysis of proteins coIP with ERMES components (baits). Fig. S5. Phylogenetic analysis of beta-barrel proteins to investigate the relationship of Porin2 of Parabasalia and Mdm10. Fig. S6. Phylogenetic analysis of T. vaginalis HSP70 chaperones. Fig. S7. Modeling of TvMmm1a homodimer. Fig. S8. Hydrophobic and polar interaction of the interface of TvMmm1a-TvMdm12 heterodimer. Fig. S9. Superposition of T. vaginalis and Z. rouxii Mmm1-Mdm12 heterotetramer. Fig. S10. Hydrophobic and polar interactions of the interface of TvMdm12-TvMmm2b. Fig. S11. Modeling of TvMmm2b-Porin2 interactions. Fig. S12-15. Raw immunoblots. [file 12915_2023_1765_MOESM2_ESM.pdf]
